# HIV-1 Proteins gp120 and Tat Promote Epithelial-Mesenchymal Transition and Invasiveness of HPV-Positive and HPV-Negative Neoplastic Genital and Oral Epithelial Cells

**DOI:** 10.1128/spectrum.03622-22

**Published:** 2022-10-31

**Authors:** Kathy Lien, Wasima Mayer, Rossana Herrera, Nicole T. Padilla, Xiaodan Cai, Vicky Lin, Rangsimon Pholcharoenchit, Joel Palefsky, Sharof M. Tugizov

**Affiliations:** a Department of Medicine, University of California-San Francisco, San Francisco, California, USA; University of Sussex

**Keywords:** epithelial neoplasia, HIV, HIV and HPV confection, HPV, HPV-16, cancer cell invasion, epithelial mesenchymal transition

## Abstract

The incidence of human papillomavirus (HPV)-associated anogenital and oropharyngeal cancer in human immunodeficiency virus (HIV)-infected individuals is substantially higher than in HIV-uninfected individuals. HIV may also be a risk factor for the development of HPV-negative head and neck, liver, lung, and kidney cancer. However, the molecular mechanisms underlying HIV-1-associated increase of epithelial malignancies are not fully understood. Here, we showed that HPV-16-immortalized anal AKC-2 and cervical CaSki epithelial cells that undergo prolonged exposure to cell-free HIV-1 virions or HIV-1 viral proteins gp120 and tat respond with the epithelial-mesenchymal transition (EMT) and increased invasiveness. Similar responses were observed in HPV-16-infected SCC-47 and HPV-16-negative HSC-3 oral epithelial cancer cells that were cultured with these viral proteins. EMT induced by gp120 and tat led to detachment of poorly adherent cells from the culture substratum; these cells remained capable of reattachment, upon which they coexpressed both E-cadherin and vimentin, indicative of an intermediate stage of EMT. The reattached cells also expressed stem cell markers CD133 and CD44, which may play a critical role in cancer cell invasion and metastasis. Inhibition of transforming growth factor (TGF)-β1 and MAPK signaling and vimentin expression, and restoration of E-cadherin expression reduced HIV-induced EMT and the invasive activity of HPV-16-immortalized anal and cervical epithelial cells. Collectively, our results suggest that these approaches along with HIV viral suppression with antiretroviral therapy (ART) might be useful to limit the role of HIV-1 infection in the acceleration of HPV-associated or HPV-independent epithelial neoplasia.

**IMPORTANCE** HPV-16-immortalized genital and oral epithelial cells and HPV-negative oral cancer cells that undergo prolonged contact with cell-free HIV-1 virions or with viral proteins gp120 and tat respond by becoming more invasive. EMT cells induced by HIV-1 in cultures of HPV-16-immortalized anal and cervical epithelial cells express the stem cell markers CD133 and CD44. These results suggest that the interaction of HIV-1 with neoplastic epithelial cells may lead to their de-differentiation into cancer stem cells that are resistant to apoptosis and anti-cancer drugs. Thus, this pathway may play a critical role in the development of invasive cancer. Inhibition of TGF-β1 and MAPK signaling and vimentin expression, and restoration of E-cadherin expression reduced HIV-induced EMT and the invasiveness of HPV-16-immortalized anal and cervical epithelial cells. Taken together, these results suggest that these approaches might be exploited to limit the role of HIV-1 infection in the acceleration of HPV-associated or HPV-independent epithelial neoplasia.

## INTRODUCTION

Human papillomavirus (HPV) has been identified as the etiological agent of nearly all anogenital carcinomas (1). Although anti-HPV vaccination strategies are effective in preventing HPV infection, the value of this approach is limited in people living with HIV, most of whom have had multiple exposures to HPV. The incidence of HPV-associated anal, cervical, and oropharyngeal cancer is 80, 22, and 6 times higher, respectively, in HIV-infected compared with HIV-uninfected individuals (2–4). HIV may also be a risk factor for HPV-negative oral, head and neck, liver, lung, testicular, and kidney cancer (5–9).

HIV may increase the risk of developing these cancers via its capacity to attenuate local and systemic immune responses to HPV. However, results from previous studies revealed that oral and genital keratinocytes exposed to cell-free HIV-1 virions and the viral envelope and transactivator proteins, gp120 and tat, respectively, induce disruptions of the epithelial tight and adherens junctions, leading to epithelial-mesenchymal transition (EMT) (10). These results suggest that direct interactions of HPV-1-infected oral or genital mucosal epithelial cells with cell-free HIV-1 and/or HIV-1 proteins gp120 and tat may induce EMT, and therefore, accelerate the development of HPV-associated malignancies. Other reports have documented the role of HIV-1 tat in inducing EMT in HPV-negative lung epithelial cancer cells as well as increased invasiveness (11). Collectively, these results suggest that HIV-1 may promote malignancy of both HPV-infected and HPV-negative neoplastic epithelial cells.

EMT is a physiologic process that regulates differentiation and acquisition of cell lineage identity during embryonic development (12). However, EMT also plays an important role in promoting neoplasia as it facilitates the growth and metastasis of invasive epithelial cancer cells (13). Cancer-associated EMT is a multistep process, which begins with the loss of apicobasal polarity in epithelial cells, followed by the loss of adherens and tight junctions, epithelial markers, and cell adhesion. During the final stages of EMT, cells acquire a spindle cell morphology and express mesenchymal markers (14–19). Cells in intermediate stages of EMT may express both epithelial E-cadherin and mesenchymal vimentin markers and thus present a hybrid phenotype; this is a critical factor contributing to the invasiveness of cancer cells (14–19).

The transforming growth factor (TGF)-β signaling pathway is the dominant canonical network that regulates cancer-associated EMT (20). TGF-β expression is activated by an AP-1 transcription factor (21) in response to MAPK signaling (22). Mature TGF-β binds to the TGFβ−R2 and initiates a signaling pathway that leads to the activation of downstream molecules, including Smad family transcription factor complexes. These complexes activate the transcriptional regulators known as Snail, Slug, and Twist1 and ultimately result in the downregulation of E-cadherin and upregulation of vimentin, fibronectin, and N-cadherin (23, 24).

Previous work from our group revealed that inhibition of MAPK and TGF-β signaling can limit the development of HIV-1-induced EMT in oral epithelial cells (10). The EMT phenotype can also be reversed in response to inhibition of vimentin and restoration of E-cadherin expression, a process known as the mesenchymal-epithelial transition (MET) (25, 26). Mechanisms underlying the induction of MET may emerge as important therapeutic targets for the prevention of cancer and/or prevent cancer cell invasion and reduce the risk of metastasis (27, 28).

Here, we investigated the contributions of HIV-1 gp120 and tat proteins and cell-free HIV-1 virions to the induction of EMT in HPV-16 immortalized anal and cervical epithelial cells as well as in HPV-infected and HPV-negative oral cancer epithelial cells. Our findings reveal that prolonged exposure of HPV-immortalized epithelial cells to HIV-1 gp120 and tat or cell-free virions promoted the development of the EMT phenotype as well as increased cell invasiveness. HIV-1 gp120 and tat proteins also increased the invasiveness of HPV-negative oral cancer cells. Collectively, the results of this work revealed that HIV-1-induced EMT in both HPV-infected and HPV-uninfected neoplastic epithelial cells may promote their migration and invasiveness, thereby leading to accelerated development of epithelial malignancy.

## RESULTS

### Characterization of the EMT in HPV-16-immortalized AKC-2 and CaSki epithelial cells.

Several of the contributions of HPV-16 E6/E7 to the development of EMT have been documented previously (14, 29–31). We began by extending these findings via an examination of the expression of the EMT markers, E-cadherin and vimentin, in HPV-16-immortalized anal AKC-2 and cervical CaSki epithelial cell lines. The HPV-16-immortalized AKC-2 cell line was previously established in our laboratory (32) from primary anal keratinocytes of HIV-infected individuals and was used in the experiments described here at passages 29 to 98. HPV-16-infected CaSki epithelial cells were originally isolated from cervical carcinoma tissue (33). The CaSki cell line has been used extensively since its introduction in 1977 and the current passage number is unknown.

**FIG 1:**
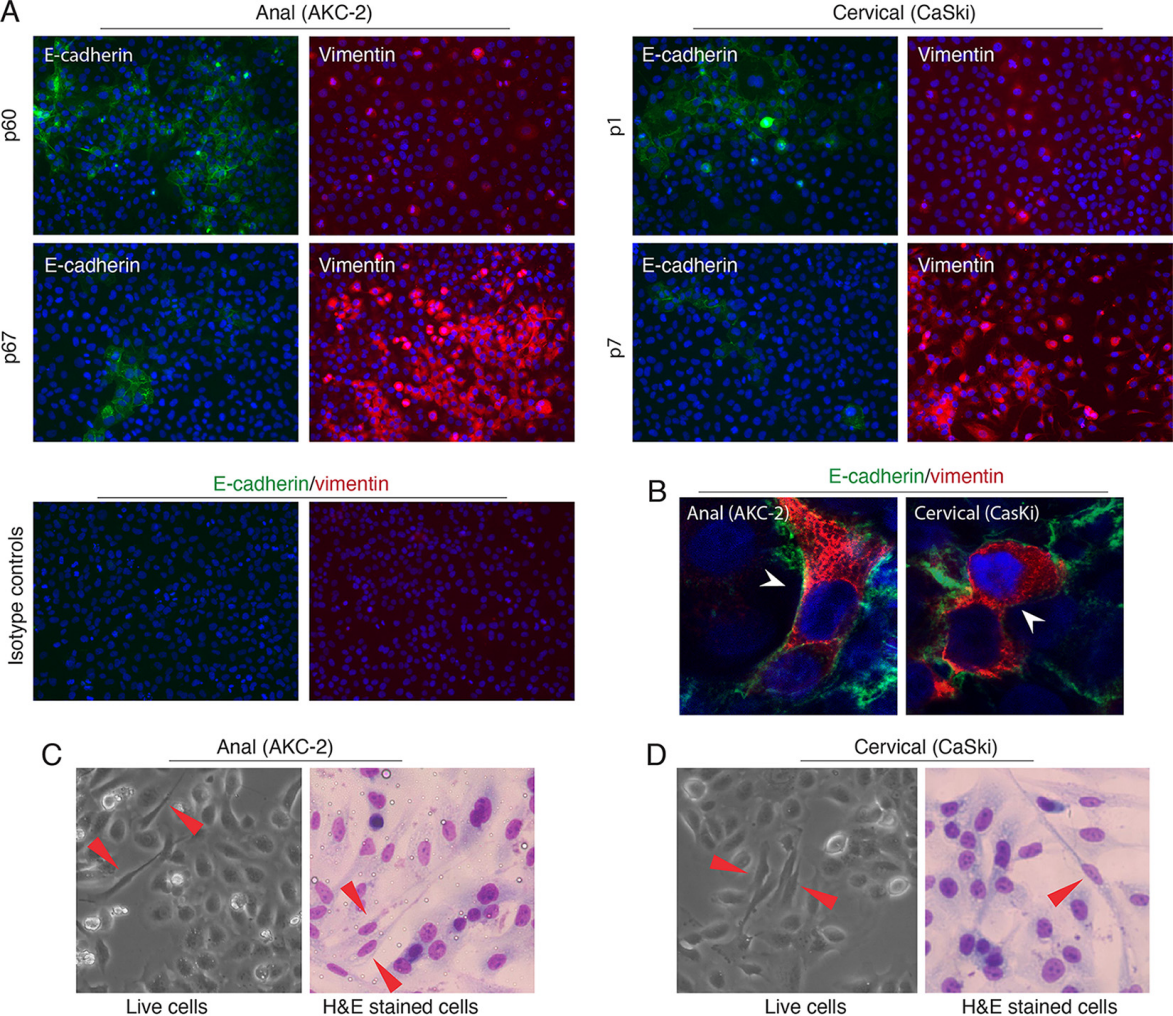
Analysis of EMT in the HPV-16-immortalized anal AKC-2 and cervical CaSki cell lines. (A) AKC-2 and CaSki cells were immunostained with anti-E-cadherin (green) and anti-vimentin (red) antibodies at passages 60 and 67 and passages 1 and 7, respectively. Cells also were stained with isotype control antibodies, which served as a negative control. Original magnification: ×400. (B) AKC-2 and CaSki cells coimmunostained with anti-E-cadherin (green) and anti-vimentin (red) antibodies. White arrowheads highlight cells that coexpress both E-cadherin and vimentin. Original magnification: ×600. (C and D) Left panels, live AKC-2, and CaSki cells were examined by phase-contrast microscopy; right panels, fixed and H&E stained AKC-2 and CaSki cells were examined by light microscopy. Red arrowheads highlight the spindle-shaped EMT cells. Original magnification: ×400. Representative images are shown.

Immunofluorescence staining revealed that ~50% of the immortalized AKC-2 cells (passage 60) expressed the epithelial EMT marker E-cadherin. We also detected the expression of the mesenchymal marker vimentin in ~20% of these cells (Fig. 1A). Interestingly, the number of cells expressing E-cadherin was substantially reduced after seven additional passages (i.e., cells evaluated at passage 67 after 2 weeks of continuous growth under similar conditions). At passage 67, E-cadherin could be detected in only ~10% of the cells. In contrast, vimentin expression increased under these conditions and was detected in 80% of the immortalized AKC-2 cells at passage 67. Similar trends were observed in experiments performed using CaSki cells; expression levels of both E-cadherin and vimentin changed after 2 weeks in culture (Fig. 1A). Co-immunostaining revealed the existence of cells that express both E-cadherin and vimentin in both the AKC-2 and CaSki lines (Fig. 1B, white arrowheads). This staining pattern reflects an intermediate stage of EMT with hybrid-phenotype cells that exhibit both epithelial and mesenchymal features.

Phase-contrast microscopy of growing live cells revealed cells with spindle-like morphology in both AKC-2 and CaSki cell cultures, indicating the presence of EMT cells (Fig. 1C and D, left panels, red arrowhead) Epithelial and mesenchymal morphology of AKC-2 and CaSki cells also was assessed by hematoxylin and eosin (H&E) staining, which revealed cells with cobblestone-like epithelial morphology or spindle-like mesenchymal morphology (Fig. 1C and D, right panels, red arrowhead).

Collectively, these results suggest that both AKC-2 and CaSki cells can undergo EMT. However, our findings revealed changes in the expression levels of both E-cadherin and vimentin during continuous growth that suggested ongoing development of epigenetic heterogeneity. To examine this possibility, AKC-2 and CaSki cells were grown continuously for 2 months; the expression and coexpression of both E-cadherin and vimentin and the development of EMT were determined every 2 weeks (Fig S1). Quantitative analysis of E-cadherin and vimentin expression revealed that the number of AKC-2 and CaSki cells expressing these markers varied significantly during continuous growth in culture. We found that an increase in the number of E-cadherin-positive cells was accompanied by a corresponding decrease in the number of vimentin-positive cells (Fig S1A). The number of cells that simultaneously expressed both E-cadherin and vimentin increased during an intermediate stage of EMT (Fig S1B) correlated with increases in the number of cells expressing both markers individually. Furthermore, the results of a quantitative analysis of cells presenting with spindle-like morphology (i.e., cells with an EMT phenotype) were consistent with the observed reduction of E-cadherin and a corresponding increase in vimentin expression (Fig S1C).

### HIV proteins tat and gp120 induce EMT in HPV-16-immortalized AKC-2 and CaSki epithelial cells.

To examine the contributions of HIV-1 to the development of EMT, HPV-16-immortalized AKC-2 and CaSki cells were cultured with HIV tat or gp120 proteins at concentrations similar to those detected *in vivo* (i.e., each at 10 ng/mL) (10, 34–37) as described in our previous publications (38–40). In parallel experiments, the same cells were treated with biologically inactive HIV tat protein with mutations in the basic arginine-rich domain and arginine-glycine-aspartic acid (RGD) motif (41–43) and heat-inactivated HIV gp120. Cells were cultured for 5 to 7 days with viral proteins. The culture medium was changed and fresh proteins were added daily.

Phase-contrast microscopy of live AKC-2 and CaSki cells treated with inactive gp120 and tat proteins, and untreated cells at 5 days posttreatment revealed few to no cells with EMT-like morphology (Fig S2). In contrast, many cells with spindle-like morphology consistent with an EMT phenotype were detected in cultures treated with active tat and gp120.

**FIG 2:**
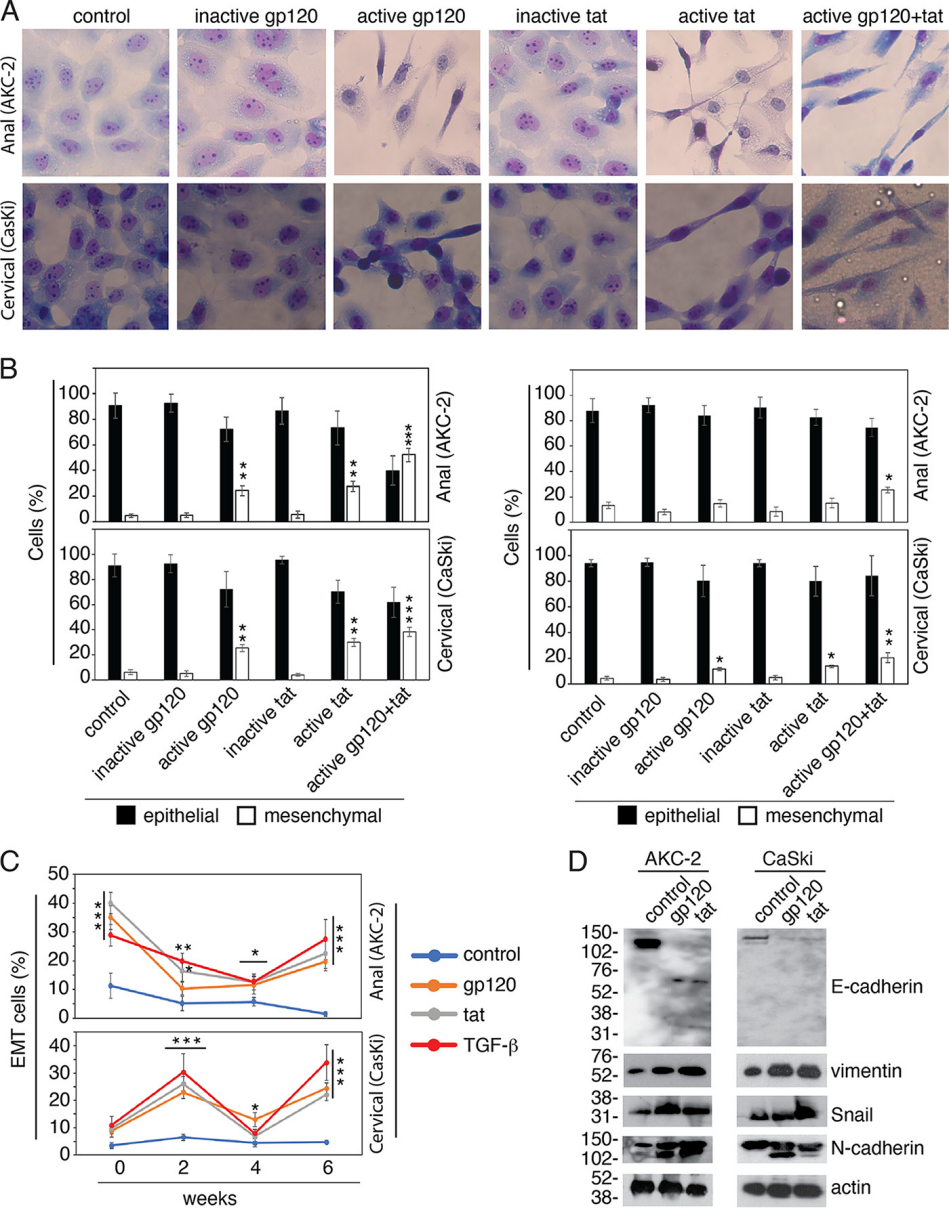
Exposure to HIV proteins gp120 and tat induces EMT in AKC-2 and CaSki cells. (A) AKC-2 and CaSki cells were untreated (control) or cultured with active and inactive forms of HIV tat and gp120 for 5 days (each at 10 ng/mL). Cells were then fixed and stained with H&E. Representative images are shown. Original magnification: ×400. (B) AKC-2 and CaSki cells grown continuously with active or inactive HIV tat or gp120 added as described in (A) were examined every 2 weeks for 4 weeks (right and left panels). The percentage of cells with epithelial *versus* mesenchymal morphology was determined in H&E-stained preparations. (C) AKC-2 and CaSki cells were grown continuously for 6 weeks. Every 2 weeks, cells were seeded in the chamber slides and treated with HIV tat, gp120, or TGF-β. The percentage of mesenchymal (EMT) cells was quantitatively evaluated in H&E-stained preparations. (B and C) Data shown are the means ± SD (*n* = 10 independent fields); *, *P* < 0.05; **, *P* < 0.01; ***, *P* < 0.001 compared with the control groups. (D) AKC-2 and CaSki cells were untreated (control) or treated with active forms of HIV tat or gp120 for 5 days. EMT induction was confirmed by microscopy and then cells were analyzed for expression of E-cadherin, vimentin, N-cadherin, and Snail by Western blotting assay. All immunoblot experiments were performed at least twice, and representative results are shown.

Multiple spindle-like cells were detected in H&E-stained preparations of both AKC-2 and CaSki cells treated with active gp120 and tat compared with control (untreated) cells at 5 days posttreatment (Fig. 2A). Quantitative analysis of H&E-stained EMT cells revealed that exposure to gp120 and tat resulted in a 20% to 25% increase in the fraction of elongated spindle-like cells along with a reduction in the fraction of cells with epithelial morphology (Fig. 2B, left panels). Approximately 3% to 5% of the cells in untreated cultures and cultures of AKC-2 and CaSki cells treated with inactive gp120 and tat displayed EMT-like morphology. Cells treated with a combination of HIV gp120 and tat proteins increased the fraction of AKC-2 and CaSki cells with EMT morphology by 52.1% and 38.3%, respectively.

However, in similar experiments, after 2 weeks of continuous growth, culture with gp120 and tat resulted in an ~8% to 10% increase in the number of EMT cells (Fig. 2B, right panels). This was not substantially higher than what was observed in untreated control cells or cells treated with inactive gp120 and tat. A significant increase in EMT cells by ~20% was detected only in AKC-2 and CaSki cells treated with a combination of gp120 + tat as well as in CaSki cells treated with active gp120 and tat proteins.

To examine HIV-1 gp120 and tat-mediated induction of EMT in AKC-2 and CaSki cells over a longer period of continuous growth, cells in culture were evaluated every 2 weeks for a total of 6 weeks. Control cells were treated with TGF-β, which is a cytokine known to induce EMT. Quantitative evaluation of H&E-stained preparations revealed that the addition of gp120, tat, or TGF-β to AKC-2 cultures induced increases in the number of EMT cells. However, these increases were not maintained during continuous cell growth. The fractions of EMT cells detected at week 0 and week 6 were higher than those detected at weeks 2 and 4 (Fig. 2C). HIV gp120 and tat-mediated increases in the number of EMT cells detected in CaSki cultures were also not stable, with lower numbers detected at weeks 0 and 4 and higher numbers at weeks 2 and 6.

To examine the expression of EMT markers by Western blotting, AKC-2, and CaSki cells were treated with gp120 and tat proteins with untreated cells serving as control. Five days later, EMT induced in the gp120- and tat-treated cells was confirmed by microscopy. Expression of E-cadherin, vimentin, Snail, and N-cadherin was then assessed by Western blotting (Fig. 2D) and protein bands were quantified by densitometry (Fig S3). Our findings revealed substantial reductions in E-cadherin and increases in vimentin expression in AKC-2 and CaSki cells treated with gp120 and tat proteins. In contrast, the untreated control cells expressed higher levels of E-cadherin and comparatively lower levels of vimentin and N-cadherin (Fig. 2D). A relatively weak immunoreactive band representing E-cadherin that was migrating with an apparent molecular mass of 76 kDa was detected in extracts of gp120- and tat-treated AKC-2 cells; by contrast, a strong E-cadherin signal at 125 kDa was detected in the untreated control cells. While N-cadherin expression was increased in gp120- and tat-treated AKC-2 cells, exposure to these HIV proteins did not significantly increase the expression of N-cadherin in CaSki cells, suggesting that N-cadherin expression may not play a critical role in EMT induction of CaSki cell (Fig S3). In gp120- and tat-treated cells, N-cadherin was detected as two bands suggesting that glycosylation of protein might be changed (44). Exposure of both AKC-2 and CaSki cells to HIV-1 gp120 and tat resulted in the increased expression of Snail, which is a transcription factor that plays critical roles in promoting the development of EMT. Collectively, these findings indicate that the prolonged exposure of HPV-16 immortalized AKC-2 and CaSki cells to HIV-1 proteins gp120 and tat proteins results in an induction of an EMT phenotype.

### HIV cell-free virions induce increases in EMT in HPV-16-immortalized AKC-2 and CaSki epithelial cell lines.

**FIG 3:**
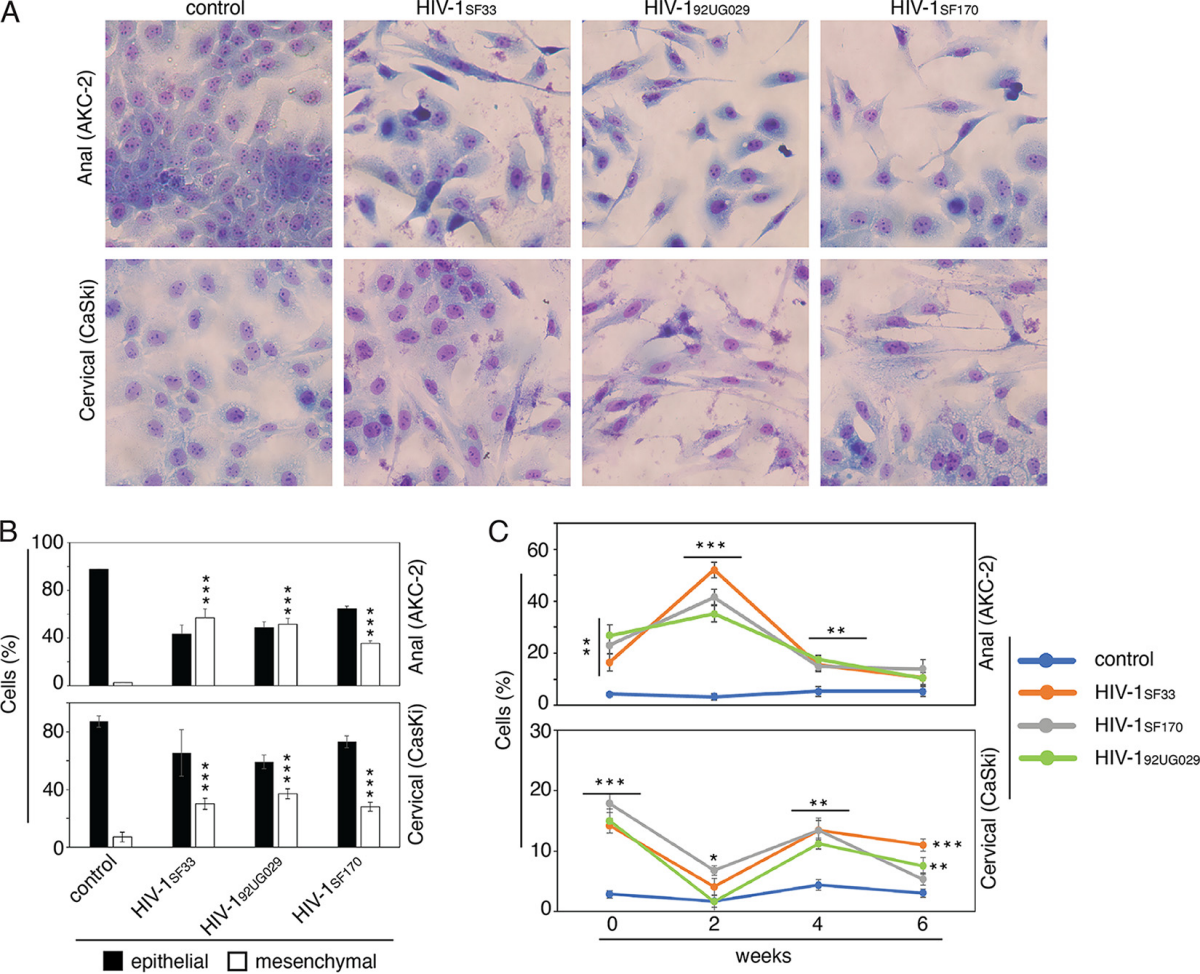
Cell-free HIV virions induce an EMT phenotype in AKC-2 and CaSki cells. (A) AKC-2 and CaSki cells that were untreated (control) or treated for 5 days with cell-free virions of dual-tropic HIV-1_SF33_, R5-tropic HIV-1_SF170_, or X4-tropic HIV-1_92UG029_ (each at 10 mg/mL) were fixed and stained with H&E. Original magnification: ×400. Representative images are shown. (B) AKC-2 and CaSki cells were untreated or treated with HIV-1 virions as described in panel A. On day 5 posttreatment, cells were immunostained with anti-E-cadherin and anti-vimentin antibodies. Cells expressing these epithelial and mesenchymal markers, respectively, were then quantitatively evaluated. Results are presented as a percentage of total epithelial and mesenchymal cells. (C) AKC-2 and CaSki cells were grown continuously and sampled every 2 weeks for a period of 6 weeks and treated with cell-free HIV-1 virions as described (A). Cells were then H&E stained and the percentage of mesenchymal cells was determined. (B and C) Data shown are the means ± SD for 10 independent fields; *, *P* < 0.05; **, *P* < 0.01; ***, *P* < 0.001 compared with untreated controls.

Given that the HIV-1 envelope protein, gp120 induces an increase in EMT in HPV-16-immortalized anal and cervical epithelial cells, this response might be replicated by exposure to HIV virions, which contain authentic gp120 in their viral membranes. To examine this possibility, AKC-2 and CaSki cells were exposed to cell-free virions (10 ng/mL of p24) of dual-tropic HIV-1_SF33_, R5-tropic HIV-1_SF170_, and X4-tropic HIV-1_92UG029_ for 7 days. H&E-stained AKC-2 and CaSki cells cultured with cell-free virions revealed that all strains induced an increase in the percentage of cells with mesenchymal morphology and a reduction in the percentage of cells with epithelial morphology in both cell lines (Fig. 3A and B). After 7 days of exposure, ~35% to 45% of the AKC-2 cells and 25% to 35% of the CaSki cells had undergone EMT and displayed a mesenchymal phenotype (Fig. 3B). Quantitative evaluation of EMT in AKC-2 and CaSki cells during 6 weeks of continuous growth revealed different patterns of virus-induced EMT in both cell lines (Fig. 3C). HIV-1-induced EMT induction in AKC-2 and CaSki cells reached maximum values at week 2 and weeks 0 and 4, respectively. Similar trends were observed in response to exposure to cell-free dual-tropic HIV-1_SF33_, R5-tropic HIV-1_SF170_, and X4-tropic HIV-1_92UG029_ strains.

To confirm its functional role in the induction of EMT, gp120 protein was preincubated with a pool of five neutralizing antibodies (b12, 2G12, F105, 39F, and ID6) or their isotype controls. AKC-2 cells were either untreated, treated with gp120 preincubated with isotype antibodies, or treated with gp120 preincubated with neutralizing antibodies. Results revealed that preincubation with antibodies that specifically target gp120 inhibited the induction of EMT approximately by ~72% compared with isotype controls, which had no impact on gp120-mediated induction of EMT in AKC-2 cells (Fig S4).

### HIV-mediated increased invasiveness of genital and oral neoplastic epithelial cells.

EMT cells have been characterized as highly motile and capable of extensive migration which promotes both invasion and cancer cell metastasis (45–48). To explore the role of the HIV-1 gp120 and tat in promoting the invasiveness of cancer cells, we examined the responses of anal AKC-2 and cervical CaSki neoplastic cells treated with HIV-1 gp120 and tat proteins in a collagen invasion assay. AKC-2 and CaSki cells were incubated with either active or inactive viral proteins, either singly or in various combinations for a period of 5 days. We performed parallel experiments with HPV-16-infected oral squamous SSC-47 and HPV-negative oral HSC-3 neoplastic epithelial cells. After treatment, cells were seeded in wells with collagen-coated inserts and cultured for an additional 24 h. Invasive cells that migrated into the collagen-coated inserts were evaluated quantitatively.

**FIG 4:**
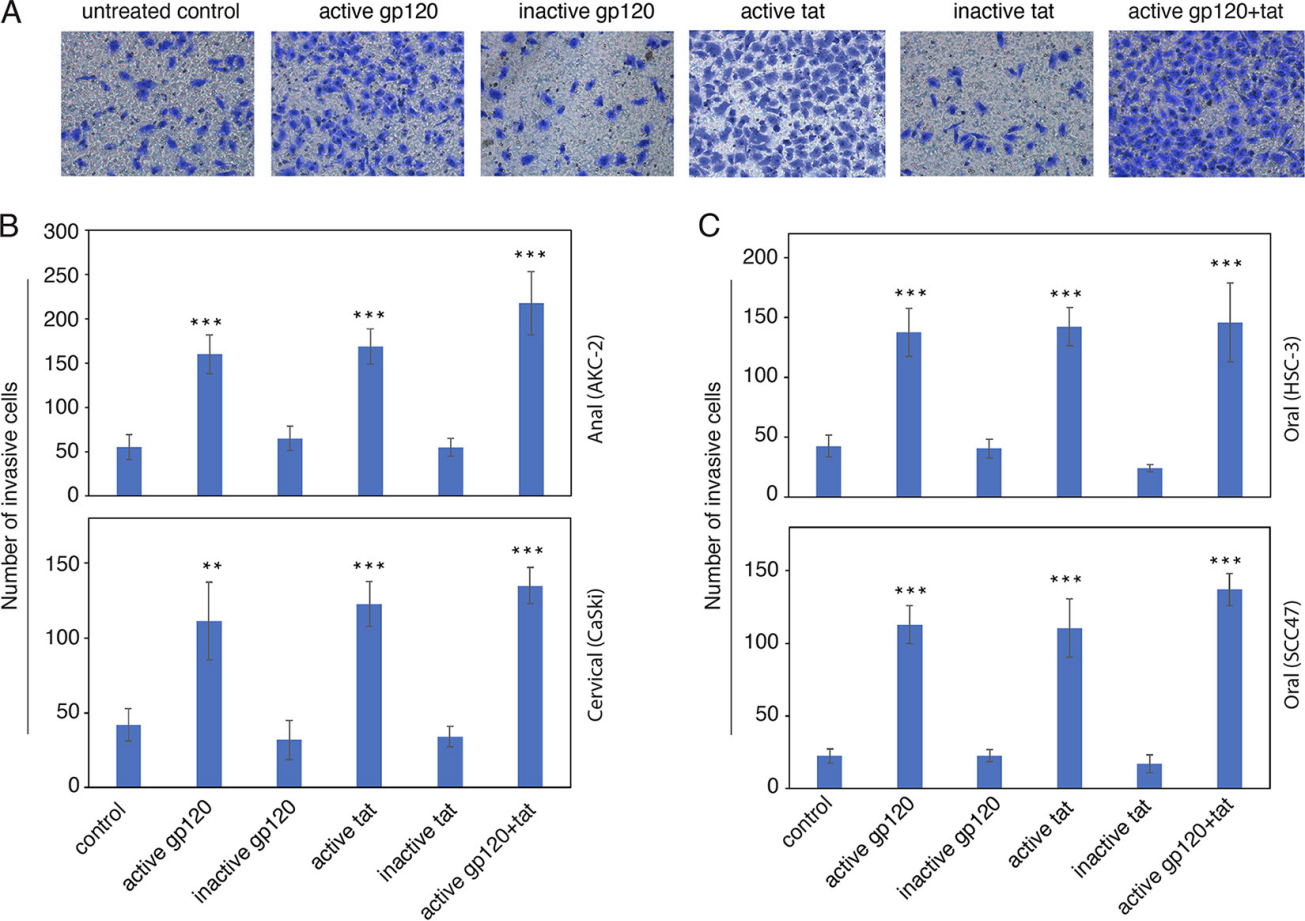
Exposure of anal, cervical, and oral neoplastic cells to HIV-1 proteins results in increased invasiveness. (A) Anal AKC-2 cells were treated with gp120 and tat, alone or in combination, for 5 days. Cells treated with inactive tat or inactive gp120 served as negative controls. EMT was confirmed by microscopy and a cell invasion assay was performed. After 24 h, cells that invaded the collagen-coated filer inserts were stained with crystal violet. Digital inverted microscopy images were obtained at an original magnification of ×100. (B) Anal AKC-2, cervical CaSki, and (C) oral HSC-3 and SCC-47 cells were treated with gp120 and tat alone or in combination as described in (A) and examined for invasive activity on collagen-coated inserts. 10 randomly selected regions were selected and invasive cells that had migrated into the collagen layer were counted. (B and C) The results presented are the average number of cells per field. Data shown are means ± SD (*n* = 10 independent regions) and are representative of two independent experiments; ***, *P* < 0.001 compared with results from inactive HIV-1 proteins (control). Results from cultures treated with cytokines were compared with untreated control groups.

The results of these experiments revealed that exposure to HIV-1 gp120 and tat proteins promotes increased invasiveness of these neoplastic epithelial cell lines by 2-fold compared with untreated cells or cells exposed to inactive gp120 or tat (Fig. 4A, B, and C). Similar trends were observed in both HPV-16-infected SSC-47 and HPV-negative HSC-3 neoplastic oral squamous epithelial cells. In these experiments, HIV-1 proteins gp120 and tat promote increased invasiveness of both HPV-16-infected and HPV-16-negative neoplastic epithelial cells (Fig. 4C).

**FIG 5:**
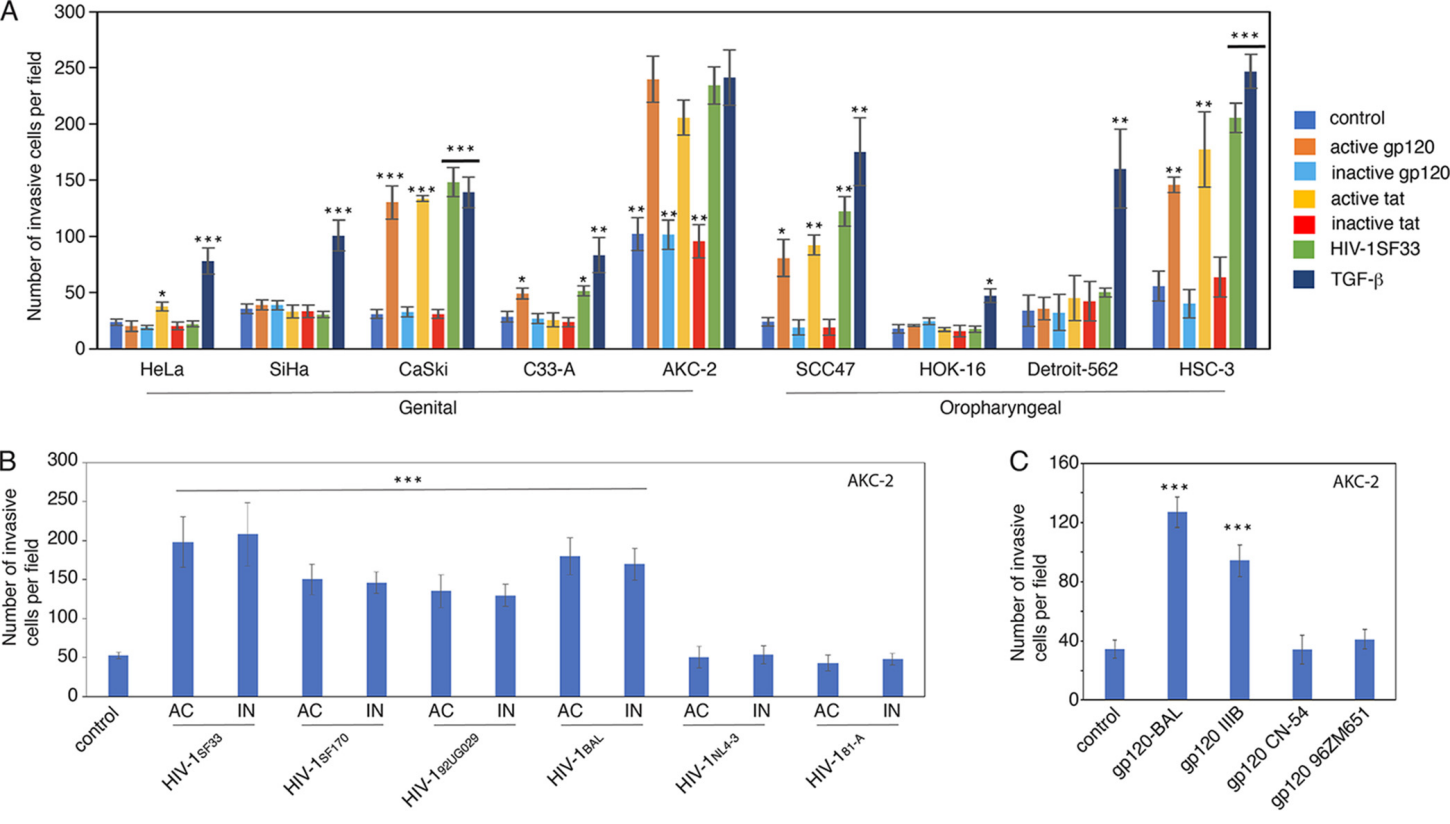
Analysis of HIV-mediated increased invasiveness in various neoplastic epithelial cells. Cervical (HeLa, SiHa, CaSki, C-33 A), anal (AKC-2), oral (SCC-47, HOK-16, HSC-3), and pharyngeal (Detroit-562) neoplastic epithelial cells were treated with inactive and active gp120 and tat, cell-free HIV-1_SF33_, and TGF-β for 5 days with untreated cells as controls. Cells were evaluated for EMT by microscopy followed by a quantitative evaluation of invasiveness using collagen-coated inserts. The results presented are the average number of invasive cells per field. (B) AKC-2 cells were treated with active (AC) or UV-inactivated (IN) cell-free dual-tropic HIV-1_SF33_, R5-tropic HIV-1_SF170_, and HIV-1_BAL_, HIV-1_81A_, X4-tropic HIV-1_92UG029_, and HIV-1_NL4-3_ strains for 5 days. EMT was confirmed by microscopy and a collagen invasion assay was performed. (C) Untreated AKC-2 cells (control) and cells treated for 5 days with gp120 from HIV-1_BAL_, HIV-1_IIIB_, HIV-1_CN-54_, and HIV-1_JR-CSF_ strains were examined in a collagen invasion assay. (A to C) Data shown are the means ± SD for three independent regions; *, *P* < 0.05; **, *P* < 0.01; ***, *P* < 0.001 compared with untreated controls.

To confirm a role for HIV-mediated increased invasiveness as a general mechanism that is characteristic of neoplastic epithelial cells, several HPV-infected and HPV-negative genital and oropharyngeal epithelial cell lines were exposed to cell-free HIV-1 virions as well as active and inactive gp120 and tat proteins. Several of these neoplastic cell lines were of genital origin, including HeLa (HPV-18-infected), SiHa (HPV-16-infected), CaSki (HPV-16-infected), AKC-2 (HPV-16 infected), and C-33 A (HPV-negative), while others were of oropharyngeal origin, including SCC47 (HPV-16-infected), HSC-3 (HPV-negative), Detroit-562 (HPV-negative), and HOK-16 (HPV-16-infected). Cells treated with TGF-β served as a positive control. The invasiveness of treated and control cells was examined using a collagen cell invasion assay. Our results revealed that gp120, tat, and cell-free virions promoted increased invasiveness of HPV-16 immortalized CaSki and AKC-2 cells (Fig. 5A). Tat treatment resulted in a slight increase in the invasiveness (35%) of HPV-18-infected HeLa cells. HPV-16 infected SiHa cells did not become more invasive when exposed to cell-free virions and viral proteins. Treatment of HPV-negative C-33 A cells with HIV gp120 or cell-free virions resulted in an ~40% increase in invasiveness. Exposure of HPV-16-infected SCC47 and HPV-negative HSC-3 cells to cell-free virions, gp120, or tat also resulted in increased invasiveness (Fig. 5A). However, no HIV-induced increase in invasiveness was observed in HPV-16-immortalized HOK-16 or Detroit-562 cells. TGF-β treatment resulted in increased invasiveness in all cell lines evaluated, consistent with our observations documenting TGF-β-mediated induction of EMT.

To determine whether the specific tropism of the cell-free HIV-1 virions plays a role in promoting invasiveness, HPV-16-immortalized AKC-2 cells were exposed to dual tropic HIV-1_SF33_, CCR5-tropic HIV-1_SF170_, HIV-1_81-A_, and HIV-1_BAL_, as well as CXCR4-tropic HIV-1_92UG029_ and HIV-1_NL4-3_ strains. Cells were treated with UV-inactivated cell-free virions in parallel experiments. The results of the collagen invasion assay revealed that exposure to cell-free virions of strains, including HIV-1_SF33_, HIV-1_SF170_, HIV-1_92UG029_, and HIV-1_BAL_, resulted in increased invasiveness, detected at 1- or 2-fold compared with untreated control cells (Fig. 5B). However, AKC-2 cells did not become more invasive in response to virions from the HIV-1_81-A_ and HIV-1_NL4-3_ strains. Similarly, no differences were detected in comparisons between cells exposed to active or UV-inactivated virions, consistent with findings that suggested a gp120-dependent mechanism.

To determine if gp120 from different viral strains could induce cell invasion, AKC-2 cells were treated with gp120 proteins from four different HIV-1 strains, including HIV-1_BAL_, HIV-1_IIIB_, HIV-1_CN-54_, and HIV-1_96ZM651_. Our results revealed that AKC-2 cells responded to gp120 proteins from HIV-1_BAL_ and HIV-1_IIIB_ strains with increased invasiveness compared to control cells (Fig. 5C). In contrast, gp120 proteins from HIV-1_CN-54_, and HIV-1_96ZM651_ strains did not promote invasiveness.

Taken together, these results indicate that cell-free HIV-1 virions, as well as viral proteins gp120 and tat, promote increased invasiveness of several genital and oral neoplastic epithelial lines, including CaSki, AKC-2, HSC-3, and SCC47. However, HIV-1-mediated increased invasiveness was not observed in SiHa, HOK-16, or Detroit-562 cells.

### Additive contributions of HIV-1 and HPV-16 to the invasiveness of AKC-2 cells.

**FIG 6:**
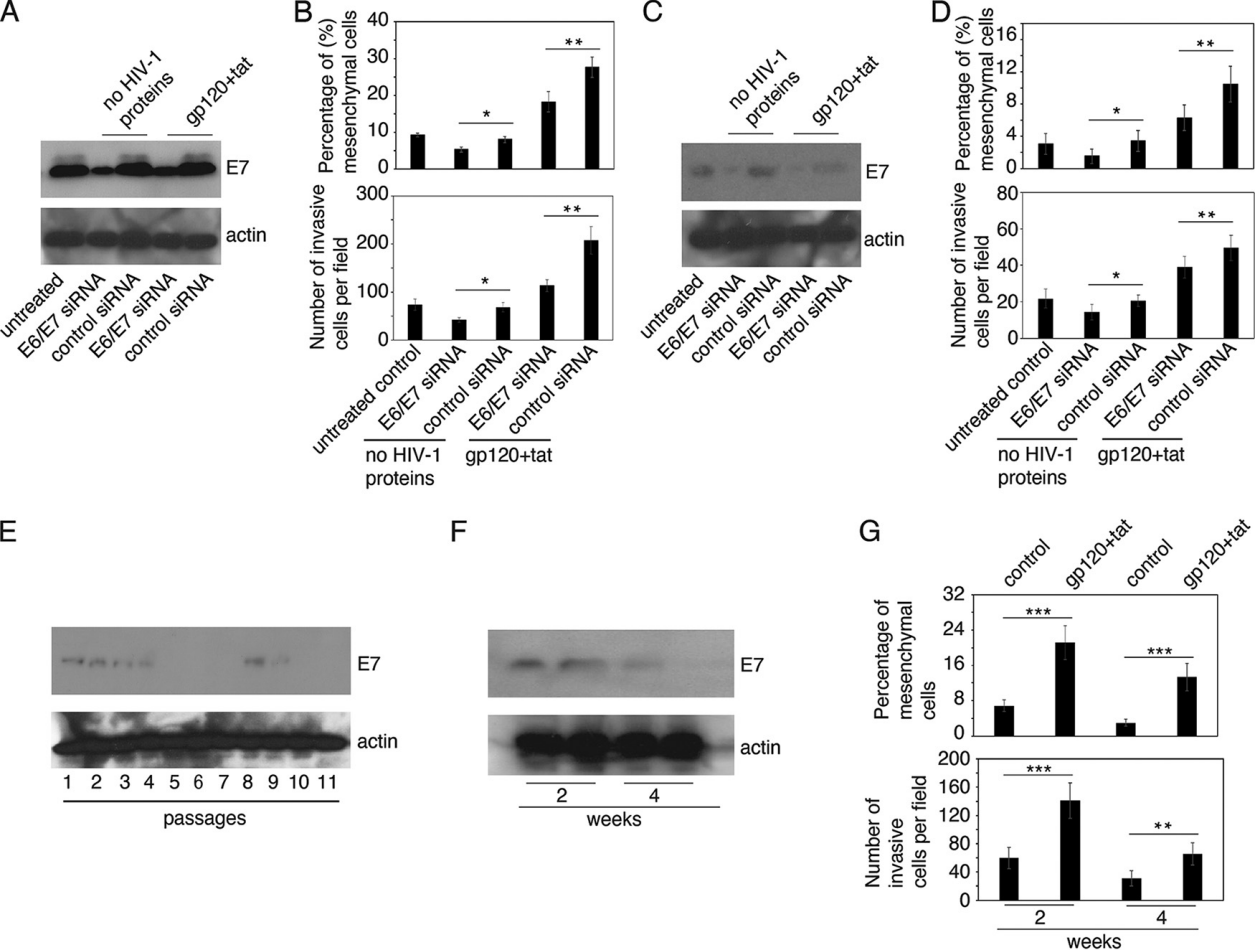
Additive impact of HIV gp120 and tat on HPV-16 E6/E7-induced EMT and cell invasiveness. (A) AKC-2 cells were transfected with HPV16-E6/E7 or control siRNAs. One set of cells was treated with HIV-1 gp120 and tat 8 h posttransfection. HPV-16 E7 expression was examined at 72 h by Western blotting. (B) In parallel experiments, AKC-2 cells were transfected with HPV16-E6/E7 or control siRNAs; after 72 h cells were either left untreated (control) or treated with gp120 and tat for the following 3 days. Untransfected and untreated cells served as controls. Induction of EMT (upper panel) and cell invasion (lower panel) were evaluated. The percentage of cells with mesenchymal morphology was determined by quantitative evaluation of H&E-stained preparations. The results of the collagen invasion assay are presented as the average number of invasive cells per field. (C and D) Similar experiments were performed in AKC-2 cells that had undergone 2.5 weeks of continuous growth. (E) CaSki cells were grown continuously for 30 days and E7 expression was examined by Western blotting after each cell passage. (F) E7 expression and (G) EMT and invasiveness were evaluated in continuously growing CaSki cells treated with HIV-1 gp120 and tat for 5 days during weeks 2 and 4 (A, C, E, and G) 10 μg of total protein was loaded in each well. (B, D, and F) Mesenchymal and invasive cells were counted in at least 10 microscopic fields. Data shown are the means ± SD from 10 independent microscopic fields; *, *P* < 0.05; **, *P* < 0.01; ***, *P* < 0.001 compared with control siRNA transfected cells.

We hypothesize that EMT induced by HIV-1 may add to the effects of HPV and may increase the invasiveness of HPV-infected cells. These interactions may increase the invasiveness of HPV-infected neoplastic cells and thus lead to an acceleration of HPV-associated malignancy. To test this hypothesis, AKC-2 cells were transfected with small interfering RNAs (siRNAs) targeting HPV-16 E6/E7. Cells transfected with a nonspecific siRNA served as a control. On day 3 posttransfection, HPV-16 E7 expression was examined by Western blotting. The results revealed diminished levels of immunoreactive E7 in AKC-2 cells transfected with the E6/E7-specific siRNA at levels that were ~70% lower than those observed in cells transfected with the control siRNA (Fig. 6A). One set of siRNA-transfected cells was treated with a combination of gp120 and tat for 5 days and then evaluated for EMT induction (Fig. 6B, upper panel) and invasiveness (Fig. 6B, lower panel). Our findings revealed that siRNA-mediated inhibition of E7 expression resulted in fewer mesenchymal cells and reduced invasiveness (both ~30%) in cultures treated with gp120 and tat compared with similarly treated cells transfected with control siRNA alone (Fig. 6B). These results suggest that ~30% of the EMT and invasiveness observed in gp120- and tat-treated AKC-2 cells were mediated by HPV-16 E6/E7.

EMT and cell invasiveness in E7 siRNA-transfected and HIV-1 gp120- and tat-treated cells was ~60% higher than in the cells transfected with E7 siRNA alone (Fig. 6B). These results indicate that gp120 and tat induced an increase in EMT and cell invasiveness specifically in cells with reduced levels of E7 expression. These findings suggest that E7 plays a role in gp120- and tat-associated induction of cell invasiveness. The number of EMT and invasive cells in gp120- and tat-treated cultures transfected with control siRNA was 3-fold higher than in those transfected with E6/E7-targeting siRNA alone and 2-fold higher than in E6/E7 siRNA transfected and gp120- and tat-treated cells. Collectively, these results indicate that expression of HPV E6/E7 and exposure to gp120 and tat have additive effects with respect to the induction of EMT and cell invasiveness.

Similar experiments were performed in AKC-2 cells after 2.5 weeks of continuous growth (Fig. 6C and D). Western blot data revealed that expression of HPV-16 E7 in nontransfected cells and control siRNA transfected cells grown for 2.5 weeks was substantially lower than under identical conditions in the first experiment. E7 was nearly undetectable in cells transfected with E6/E7 siRNA. After 2.5 weeks of continuous growth, we observed ~90% inhibition of E7 expression in siRNA-transfected ACK-2 cells compared with those transfected with control siRNA. EMT and invasiveness of the control cells in this second trial were also approximately 3-fold lower than observed in control cell cultures in the first experiment, possibly due to the reduced expression of E7.

We also examined the additive effects of HIV-1 gp120 and tat proteins and HPV-16 E7 in EMT induction and invasiveness of CaSki cells. First, HPV-16 E7 expression was examined in CaSki cells undergoing continuous growth in culture for 30 days (11 passages). Western blotting revealed low levels of E7 expression during passages 1 to 4 and 8 to 9; no E7 was detected at any other time point. These findings suggest that HPV-16 E7 is not stably expressed in CaSki cells and may lost during continuous cell growth.

To examine the additive effect of HIV-1 gp120 and tat and HPV-16 E7, CaSki cells were treated with gp120 and tat at week 2 and week 4; cells were then examined simultaneously for E7 expression (Fig. 6F), and EMT and invasiveness (Fig. 6G). Western blotting revealed E7 expression at weeks 1 and 2; however, expression of this protein was drastically reduced at week 3 and was lost by week 4 (Fig. 6F). Quantitative analysis of EMT and invasiveness revealed that treatment with gp120 and tat resulted in 2-fold increases in EMT and invasiveness at week 2 compared with untreated controls (Fig. 6G), corresponding to detectable E7 expression at this time point. At week 4, the levels of EMT and invasiveness of both untreated and HIV-1 gp120- and tat-treated CaSki cells were significantly lower than those observed at week 2. This finding correlated with reductions and/or complete loss of E7 expression. However, at week 4, levels of HIV-1 gp120- and tat-induced EMT and invasiveness were higher than those observed in untreated control cells. These results indicate that gp120 and tat can promote increased EMT and invasiveness in the absence of E7 expression, albeit to levels that were ~50% less than those detected in cells expressing this protein.

These findings indicate that variations observed with respect to EMT and cell invasiveness in continuously growing AKC-2 and CaSki cells may be directly parallel and dependent on the expression of HPV-16 E6/E7. Nevertheless, the observed gp120- and tat-mediated increases in EMT and cell invasiveness, with or without expression of HPV E7, were associated with similar outcomes in both cells. The additive effects of HIV-1 gp120 and tat and HPV-16 E6/E7 on the induction of EMT and cell invasiveness were observed in both cell lines. Taken together, these results suggest that HIV- and HPV-mediated induction of EMT and cell invasiveness may play critical roles in promoting HPV-16-associated neoplastic disease.

### EMT cells generated in response to HIV-1 gp120 and tat can detach, reattach, and develop hybrid-type cells that express stem cells markers CD133 and CD44.

**FIG 7:**
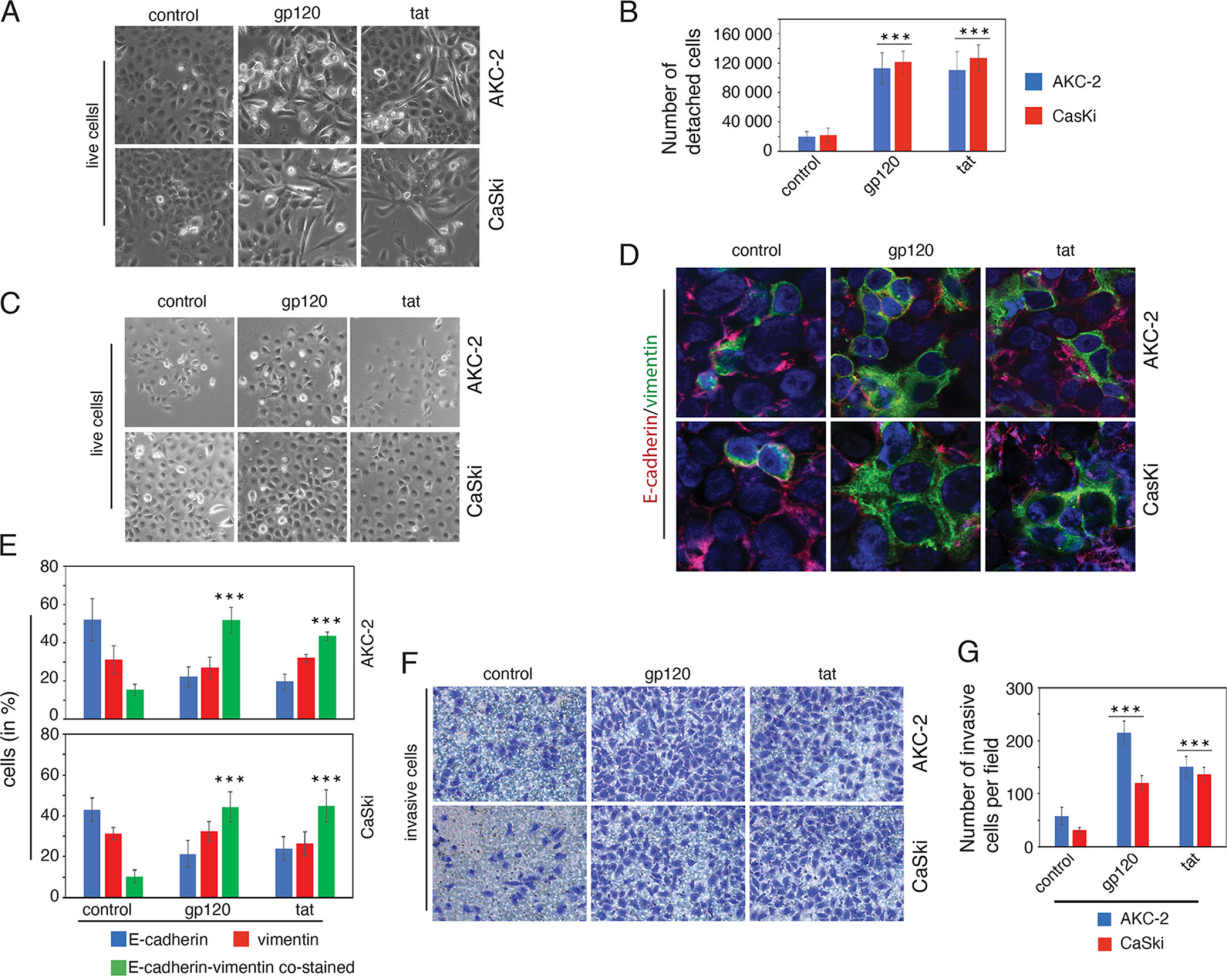
HIV-1 gp120 and tat treatment of AKC-2 and CaSki cells promote detachment of EMT cells that coexpress E-cadherin and vimentin upon reattachment. (A) AKC-2 and CaSki cells were left untreated (control) or treated with HIV-1 gp120 or tat for 5 days. Live cells were examined by phase-contrast microscopy at a magnification of ×100. Representative images are shown. (B) On day 5 posttreatment, weakly attached and floating AKC-2 and CaSki cells were collected from six-well plates and counted. These values are presented as numbers of cells per well. (C) Untreated control cells and treated AKC-2 and CaSki cells were seeded into eight-chamber slides and grown in regular media without gp120 or tat treatment. Three days later, reattached live cells (passage 0) were examined by phase-contrast microscopy at a magnification of ×100. Representative images are shown. (D) Reattached AKC-2 and CaSki cells that originated from untreated control cells or cells treated with gp120 or tat were coimmunostained at passage 0 with anti-E-cadherin (red) and anti-vimentin (green). The cell nuclei are shown in blue at a magnification of ×400. (E) Quantitative evaluation of reattached AKC-2 and CaSki cells that express E-cadherin, vimentin, or both markers. (F and G) Invasiveness of the reattached AKC-2 and CaSki cells (passage 2) was evaluated quantitatively with a collagen invasion assay. (G) The results of the collagen invasion assay are presented as the number of invasive cells per field at a magnification of ×100. (B, E, and G) Data shown are the means ± SD for 10 independent fields; *, *P* < 0.05; **, *P* < 0.01; ***, *P* < 0.001 compared with untreated controls.

Cancer cells that have undergone EMT may lose their adhesive functions; these cells can detach from the culture substratum and float in the culture media (49). We have observed both floating and weakly adherent AKC-2 and CaSki cells that may exhibit an EMT phenotype. To examine the relationship between HIV-1 induced EMT and the generation of detached cells, AKC-2 and CaSki cells were treated with HIV-1 gp120 and tat for 5 days; untreated cells served as controls. On day 5, phase-contrast microscopy revealed that exposure to HIV-1 gp120 and tat resulted in an increase in the number of live EMT cells compared with untreated controls (Fig. 7A). Weakly adherent cells were detached from the culture substratum by gently shaking the flask, and floating cells were collected and counted. Our results revealed that the number of live floating cells was approximately 5-fold higher in gp120- and tat-treated cultures than in untreated controls (Fig. 7B). These cells were then seeded into chamber slides and examined 3 days later by live phase-contrast microscopy (Fig. 7C). Our results revealed that the floating cells were capable of reattachment to the culture wells. Cells were immunostained with anti-E-cadherin and anti-vimentin; cells that expressed E-cadherin alone, vimentin alone, or both E-cadherin and vimentin were detected by confocal microscopy (Fig. 7D). Quantitative evaluation of these data revealed that treatment with gp120 or tat resulted in 5- and 4-fold increases in the number of E-cadherin and vimentin coexpressing cells, respectively, compared with control cells (Fig. 7E). These results indicate cells that were initially detached and then reattached tend to exhibit a hybrid phenotype.

To examine the invasiveness of the reattached cells, floating AKC-2 and CaSki cells were collected from gp120- or tat-treated cells and untreated controls and were seeded into new flasks. These cells were examined for invasiveness after 5 days (Fig. 7F). Our results revealed that gp120- and tat-treated cells were 3- and 2.5-fold more invasive than the untreated controls (Fig. 7F and G). These results suggested that cells that became detached cells in response to gp120 and tat treatment were capable of generating a new cell population with high levels of E-cadherin and vimentin coexpression and higher levels of invasive activity.

**FIG 8:**
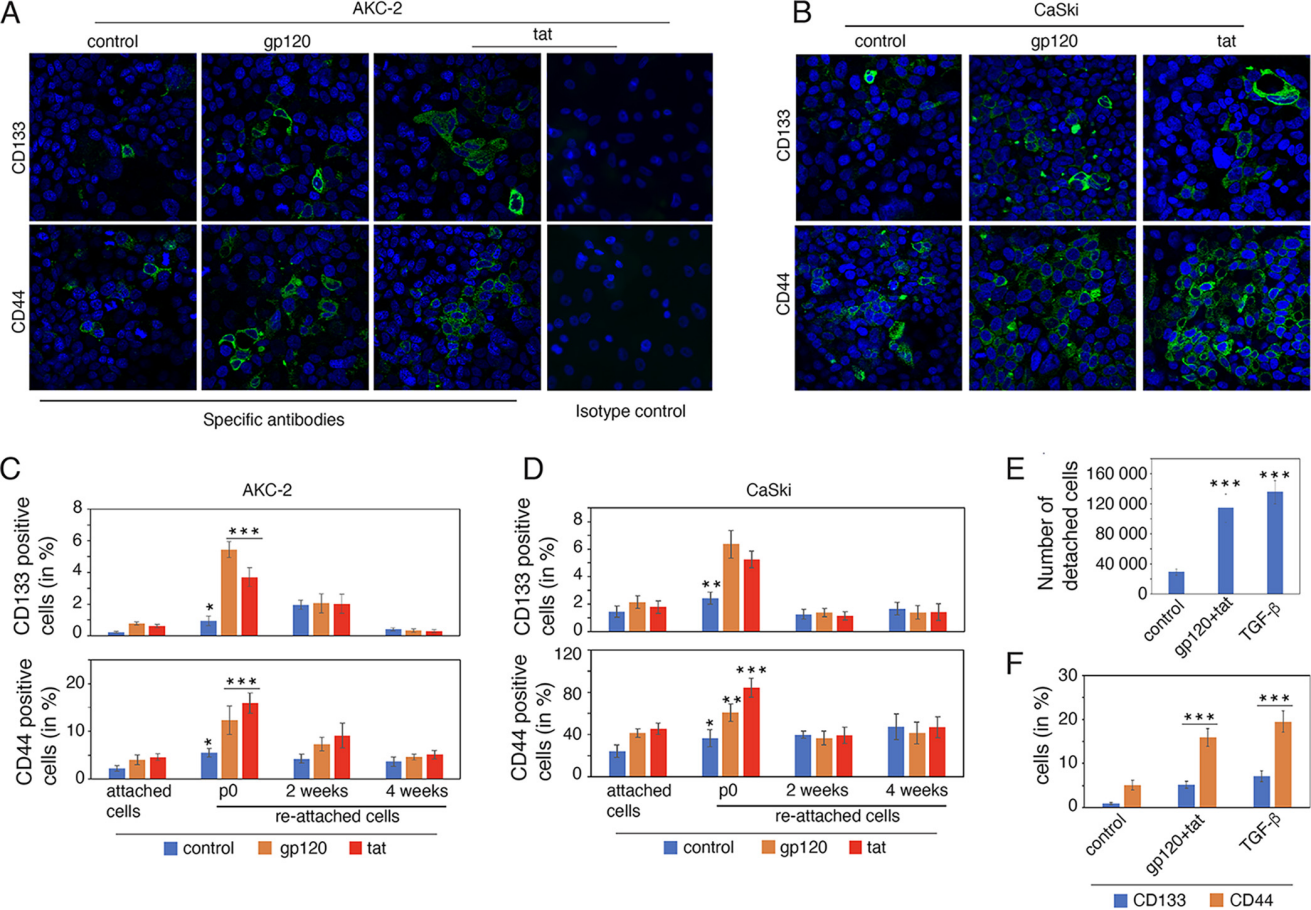
Reattached EMT cells express cancer stem cell markers CD133 and CD44. (A and B) Detached cells were collected from untreated control cultures and AKC-2 and CaSki cultures treated with gp120 or tat. These cells were seeded into chamber slides. Cells that had reattached after 5 days were immunostained with anti-CD133 and anti-CD44 (both in green). The cell nuclei are shown in blue at a magnification of ×200. (C and D) Attached AKC-2 and CaSki control cells and cells treated with gp120 or tat on day 5 (attached cells) and reattached cells that originated from floating cells at passage 0 and later passages (at weeks 2 and 4) were immunostained with anti-CD133 and anti-CD44; antigen-infected cells were evaluated quantitatively. (E and F) AKC-2 cells were treated for 5 days with gp120, tat, or TGF-β, and detached cells were collected, counted, and (E) seeded into chamber slides. Three days later, cells were immunostained with anti-CD133 and anti-CD44; marker-infected cells were counted (F). (C, D, E, and F) Data shown are the means ± SD from counts of at least 10 microscopic fields; *, *P* < 0.05; **, *P* < 0.01; ***, *P* < 0.001 compared with untreated controls.

E-cadherin and vimentin coexpressing malignant hybrid cells may also express stem cell markers (15–19, 50, 51). To evaluate this possibility, floating AKC-2 and CaSki cells were collected from gp120- or tat-treated and untreated controls. Floating cells were seeded into chamber slides. Reattached cells were examined for expression of stem cell markers CD133 and CD44. Our results revealed an increase in the number of gp120- and tat-treated AKC-2 and CaSki cells that expressed CD133 and CD44 compared with untreated control cells (Fig. 8A and B). Quantitative analysis of gp120- and tat-treated or control cells expressing immunoreactive CD133 and CD44 was performed on the following three subsets: (i) attached cells at day 5 posttreatment, (ii) reattached cells after 5 days (passage 0), and (iii) reattached cells after 2 and 4 weeks of continuous growth. Our results revealed that ~0.2% and 5% of the untreated AKC-2 control cells were CD133- or CD44-positive cells, respectively (Fig. 8C). Expression of these stem cell markers increased ~2-fold in response to treatment with gp120 and tat. We observed a 3- to 5-fold increase in CD133- and CD44-expressing cells among treated cells that were initially floating and then reattached compared with untreated cells. A similar trend was observed in experiments involving reattached CaSki cells that were collected from gp120- and tat-treated cultures compared with untreated control cells (Fig. 8D). The number of CD133- and CD44-expressing cells in gp120- and tat-treated cultures was 2-fold higher than that detected in untreated controls. However, after 2 to 4 weeks of continuous growth, we observed a gradual reduction in the number of gp120- and tat-treated reattached cells that expressed CD133 and CD44; eventually, their numbers were not significantly different from those detected in untreated control cultures. In other words, our results suggest that gp120- and tat-mediated induction of detached and reattached cells and the associated expression of stem cell markers disappeared from this cell population over time.

To determine whether TGF-β was also capable of inducing detachment and CD133 expression, AKC-2 cells were treated with TGF-β or HIV-1 gp120 and tat for 5 days. Comparable numbers of detached cells from TGF-β or HIV-1 gp120 and tat-treated cultures were obtained (Fig. 8E). Detached cells were seeded into chamber slides to facilitate reattachment. Immunostaining of reattached cells with anti-CD133 revealed that cultures treated with either TGF-β or HIV-1 gp120 and tat treatment generated ~4.5% CD133-positive cells after reattachment (Fig. 8F). The number of cells expressing CD44 in TGF-β or HIV-1 gp120-treated cultures was 3- to 4-fold higher than in untreated controls.

Taken together, our findings reveal that AKC-2 and CaSki cell populations maintain comparatively small fractions (~0.5% to 5%) of cells that are detached and floating due to the loss of their adhesive properties; the number of detached and floating cells increased 4- to 5-fold in response to treatment with gp120 and tat. Most of the floating cells that were reattached to the new flask exhibit a hybrid EMT phenotype as they coexpress E-cadherin and vimentin as well as stem cell markers CD133 and CD44.

### Inhibition of HIV-1-induced EMT in AKC-2 cells limits their invasive activity.

**FIG 9:**
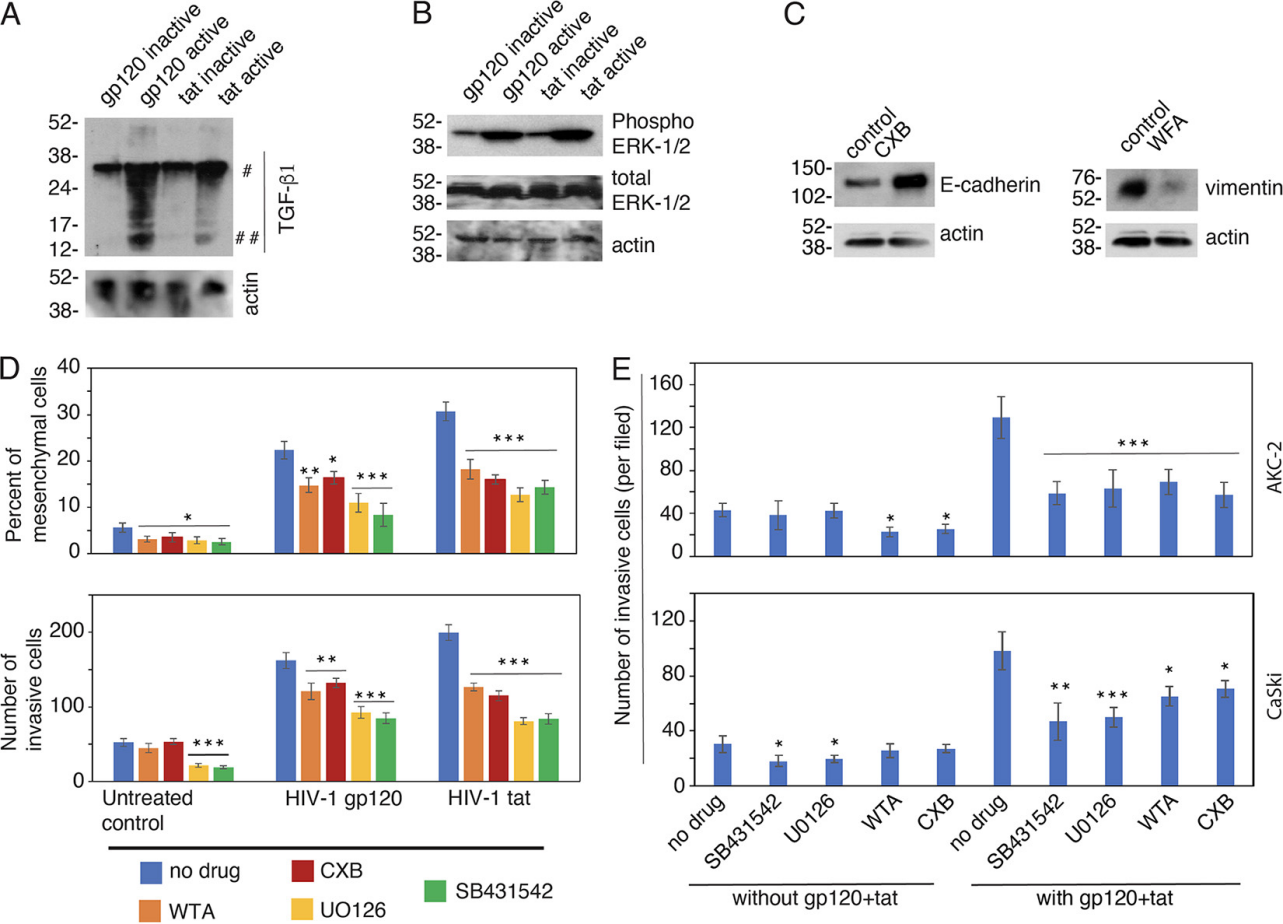
Inhibition of TGF-β1 and MAPK signaling and vimentin expression, and restoration of E-cadherin reduce HIV-mediated induction of the EMT phenotype in AKC-2 and CaSki cells. (A and B) AKC-2 cells were treated for 5 days with active or inactive forms of gp120 or tat. Cell extracts were phosphorylated and total ERK1/2, TGF-β1, and actin were detected by Western blotting. (A) # precursor and ## mature active forms of TGF-β1 are as shown. (The blots shown are representative of two independent experiments). (C) AKC-2 cells were treated with celecoxib (CXB; cyclooxygenase-2 inhibitor) or withaferin A (WFA, vimentin inhibitor) for 5 days. Untreated cells served as controls. Extracts were prepared and Western blots were performed to detect E-cadherin and vimentin expression. (D) AKC-2 cells were treated gp120 or tat for 5 days in the presence or absence of inhibitors of MAPK (UO126), TGF-β1 (SB431542), vimentin (WFA), or cyclooxygenase-2 (CXB); cells that were not treated with proteins or inhibitors served as controls. On day 5, the percentage of mesenchymal cells (upper panel) and invasiveness (lower panel) were examined quantitatively. (E) AKC-2 and CaSki cells were treated with gp120 or tat alone or in combination for 5 days in the presence or absence of UO126, SB431542, WFA, or CXB. Cells that were treated with inhibitors in the absence of gp120 or tat served as controls. Cell invasiveness was assessed on day 5. (D and E) The data shown are representative of three independent experiments and are presented as means ± SD of 10 independent fields; *, *P* < 0.05; **, *P* < 0.01; ***, *P* < 0.001 compared with cells that were or were not treated with inhibitors.

We have shown that HIV-mediated activation of TGF-β1 and ERK1/2 signaling pathways play a critical role in the induction of EMT in primary oral and genital epithelial cells (10). To determine whether HIV-induced EMT in HPV-16-immortalized epithelial cells also requires the activation of TGF-β1 and ERK1/2 signaling pathways, AKC-2 cells were treated with active or inactive gp120 and tat proteins for 5 days. Cell extracts were prepared and examined by Western blotting for evidence of TGF-β1 and ERK1/2 activation. We detected the cleaved form of mature TGF-β1 protein in extracts of AKC-2 cells that were exposed to active gp120 or tat; little to no cleaved TGF-β1 protein was detected in cells treated with the inactive form of these proteins (Fig. 9A). We also detected increases in phosphorylated ERK1/2 in cells from cultures treated with the active forms of both gp120 and tat (Fig. 9B). These data demonstrate that HIV gp120 and tat can promote activation of TGF-β1 and MAPK signaling, which are critical pathways in AKC-2 cells that are associated with the induction of the EMT phenotype.

Inhibition of vimentin and restoration of E-cadherin expression may lead to normalization of epithelial morphology, i.e., to the reverse, mesenchymal-epithelial transition (MET) (25, 26, 52, 53). To induce the MET phenotype, AKC-2 cells were treated with nontoxic concentrations of celecoxib (CXB) and withaferin A (WFA) (Fig S5), which upregulates the transcription of E-cadherin and induces the degradation of vimentin, respectively (54–56).

The expression of E-cadherin and vimentin was assessed in extracts from AKC-2 cells by Western blotting. Our results revealed that treatment of AKC-2 cells with CXB substantially increased E-cadherin expression compared with untreated controls (Fig. 9C). Treatment of AKC-2 cells with WFA resulted in reduced vimentin expression compared with control cells. Collectively, these findings indicate that CXB- and WFA-mediated restoration of E-cadherin and inhibition of vimentin expression induces MET in HPV-16-immortalized AKC-2 cells.

EMT and invasive activity of AKC-2 cells treated with gp120 and tat in the presence or absence of inhibitors for 5 days was examined. Our results revealed that exposure to inhibitors of TGF-β and MAPK signaling as well as treatment with WFA and CXB reduced EMT in untreated control cells 45% to 50% (Fig. 9D, upper panels). However, treatment with WFA and CXB did not reduce the invasive activity of control cells. In contrast, inhibitors of TGF-β and MAPK have a marked impact on invasive activity. While all four inhibitors blocked gp120- and tat-induced EMT (Fig. 9D, upper panels) and cell invasion (Fig. 9D, lower panels), the reductions in EMT and invasive activity mediated by TGF-β and MAPK inhibitors were stronger (50% to 70%) than those in response WFA and CXB (30% to 40%).

In our final experiments, AKC-2 and CaSki cells were treated for 5 days with gp120 and tat in the presence or absence of the aforementioned four inhibitors. Cells were then evaluated in a collagen invasion assay. Our results revealed that treatment with WFA and CXB resulted in a 30% reduction in spontaneous AKC-2 invasive activity, although they had no impact on the invasion of control CaSki cells (Fig. 9E). However, inhibitors of TGF-β and MAPK signaling resulted in a 30% reduction in the invasive activity of CaSki cells; by contrast, they had no significant impact on AKC-2 cell invasion. Treatment with the four inhibitors resulted in a 45% to 50% reduction in the invasive activity of gp120- and tat-treated AKC-2 cells. Inhibitors of TGF-β and MAPK signaling had a stronger (50%) effect than WFA and CXB (30%) on CaSki cell invasion induced by gp120 and tat.

These findings indicate that inhibition of MAPK and TGF-β signaling together with degradation of vimentin and restoration of E-cadherin expression serve to reduce the invasiveness of HPV-16 immortalized anal and cervical epithelial cells treated with HIV-1 gp120 or tat. These mechanisms are also effective in untreated control cells that express HV-16 E6/E7.

## DISCUSSION

The findings presented in this work reveal that prolonged interaction of HIV-1 proteins gp120 and tat and cell-free HIV-1 virions with HPV-16-immortalized anal, cervical, and oral epithelial cells resulted in an increase in their EMT and invasiveness. These results suggested that the direct interaction of HIV with HPV-16-infected neoplastic cells may potentiate HPV-associated malignancy. Among our most interesting findings, our results revealed that HIV-1 gp120- and tat-induced EMT cells with an intermediate phenotype express stem cell markers CD133 and CD44, which may play a critical role in the formation of cancer stem cells (CSCs) that are responsible for the development of invasive cancer. These findings may explain at least in part the higher incidence of HPV-associated genital and oral cancer in HIV-infected individuals compared with those who are not infected with HIV. We also showed that exposure to HIV-1 gp120 increased the invasiveness of HPV-negative neoplastic oral epithelial cells; these results suggest that HIV-1 may also promote malignancy in the absence of HPV infection.

The number of EMT cells detected in both AKC-2 and CaSki lines underwent substantial change during 2 months of continuous growth in culture. Furthermore, we found that these changes paralleled variations in the number of cells expressing E-cadherin and vimentin. This is consistent with findings reported previously that documented the extensive heterogeneity of HPV-18-infected HeLa cells (57–59). The transcriptomes, proteomes, and protein turnover profiles of these HeLa variants underscored progressive divergence that developed over the course of 50 successive passages under uniform conditions (57). Single-cell analysis revealed that only a limited fraction (3.2% to 6.1%) of HeLa cells in a given population are immortal and capable of self-renewal (59). Likewise, only a small proportion of these HeLa cells (0.16%) express the stem cell marker CD133, although this fraction was substantially higher (3.64%) in sorted cells (60). These CD133-positive immortal HeLa cells may be CSCs (61, 62) that are capable of generating heterogeneous cell populations with distinct genetic and epigenetic profiles.

The continuous change in the fraction of cells expressing E-cadherin and/or vimentin, as well as the fraction of cells with epithelial, hybrid, or mesenchymal phenotypes suggests that the AKC-2 and CaSki cell lines are also heterogenous and likewise may contain only a small number of immortal stem cells that ultimately generate genetically and phenotypically distinct cell populations. Our findings revealed that a subpopulation of AKC-2 and CaSki cells express stem cell markers CD133 and CD44. Of note, results from previous studies have revealed that CD133-infected CaSki cells have features consistent with stem cells (63).

Continuous and ongoing changes in the expression of epithelial and mesenchymal markers may be the direct result of ongoing genetic and epigenetic changes in these cells, similar to what was reported for HeLa and other neoplastic cell lines. It is also possible that the removal of the floating cells with stem cell properties by the washing step required for effective trypsinization of the cell monolayer may contribute to the generation of heterogeneous cell populations. Changes in the number of floating cells from one passage to the next may differentially enrich the culture with cells that express stem cell markers, a process that may ultimately lead to the generation of different cell populations and differential regulation of epithelial and mesenchymal markers. Cell heterogeneity is a well-known feature of invasive cancer *in vivo*; the high degree of heterogeneity observed among epithelial tumor cells is understood to be critical for the progression of malignancy (64).

The addition of cell-free HIV-1 virions or viral proteins gp120 and tat resulted in a significant increase in EMT in cultures of HPV-16-immortalized AKC-2 and CaSki cells. Of note, the HIV-mediated increase in EMT could not always be detected during a 6-week period of continuous cell growth. Our results suggest that this was mainly due to changing E-cadherin and vimentin expression patterns. Unstable expression of HPV-16 E6/E7 paralleled the variation in the numbers of EMT cells detected in cultures of AKC-2 and CaSki cells. These results suggested that changes in the expression of HPV-16 genes in continuously growing cells may lead to variations in the number of EMT cells as well as overall invasiveness.

There may be other genetic and epigenetic changes taking place in both AKC-2 and CaSki cell cultures, including variations in the expression levels of epithelial receptors that interact with gp120 and tat. Most mucosal epithelial cells express heparan sulfate proteoglycans (HSPGs) and galactosyslceramides (GalCers) that serve as receptors for HIV-1 gp120 (65–68). HIV-1 tat binds to α5β1, α5β3, and αvβ3 integrins that are also expressed by epithelial cells (69–72). Ongoing genetic and phenotypic changes detected in AKC-2 and CaSki cells may be directly related to changes in HSPG, GalCer, and integrin expression that may have an impact on gp120 and tat binding and may modulate the functional impact of the HIV viral proteins.

Our findings revealed that many cervical and oral neoplastic cell lines become more invasive upon exposure to cell-free HIV-1 virions or gp120 and tat proteins, and showed that virus and viral proteins induce the increased invasiveness of both HPV-16-infected AKC-2, CaSki, and SCC47 and HPV-negative HSC-3 cell lines. Interestingly, neither virus nor viral proteins induced an increase in the invasive activity of HPV-16-infected SiHa or HOK-16 cells or HPV-18-infected HeLa cells. This discrepancy might be due to the absence of critical epithelial receptors or signaling molecules in the latter set of cell lines. Differential expression of cell surface receptors and signaling molecules may also be a result of ongoing genetic and epigenetic changes in neoplastic epithelial cells; these changes may lead to downregulation of HSPG, GalCer, and integrin expression which are critical mediators of HIV-1 gp120 and tat binding (65–72). Induction of EMT in AKC-2 and CaSki cells in response to HIV-X4, HIV-R5, and dual-tropic HIV-1 viruses suggests that viral tropism is not a critical mediator of this process. However, our observation that two of four recombinant gp120s and two of six virus strains were unable to induce EMT of AKC-2 cells suggests that some HIV-1 strains may have altered gp120 that cannot bind to its designated epithelial receptors.

HIV-1 gp120 and tat induction of EMT resulted in reduced cell adhesion and detachment of HPV-16-immortalized AKC-2 cells with hybrid phenotype with mixed epithelial (i.e., adhesive) and mesenchymal (i.e., migratory) properties (14, 16, 49). *In vivo*, these cells may migrate as clusters to form circulating tumor cells that generate new metastases at distant locales (16, 17). Neoplastic cells with a hybrid phenotype may be resistant to apoptosis and with more potential for invasion than cells with a full mesenchymal phenotype (14, 73–75).

We detected increased expression of both CD133 and CD44 in hybrid-phenotype AKC-2 and CaSki cells treated with gp120 and tat. These cells may have been generated by the de-differentiation of EMT cells and may function as stem cells (15–19, 50, 51). CSCs play critical roles in promoting the metastasis of tumor cells and developing drug resistance (76–80). HIV-1 gp120 and tat may promote the formation of CSCs within the HPV-16 infected neoplastic epithelia; these cells may accelerate the development of HPV-associated malignancy *in vivo*.

HPV-associated cancers have not declined in the HIV-infected population in the era of antiretroviral therapy (ART), and normalization of the immune response did not reduce the incidence of HIV/HPV-associated cancers (4). ART may not fully eliminate HIV from mucosal immune cells (38) potentially because of the limited penetration of these drugs into solid tissues (81–84). HIV-1 infected CD4^+^ lymphocytes, macrophages, and Langerhans cells were detected in the anogenital and oropharyngeal mucosal epithelia of HIV-infected individuals receiving ART (38). Furthermore, HIV-1 tat was continuously released from infected cells even under an ART regimen (83). Thus, HIV replication within the anogenital and oral mucosa may induce EMT of HPV-infected epithelial cells as well as a CSC phenotype, thereby promoting the HPV-associated neoplastic process even in HIV-infected individuals receiving ART.

Our results also revealed that HIV-1 gp120 and tat proteins promoted the invasiveness of HPV-negative oral cancer HSC-3 cells. These findings suggest that HIV-1 may also promote malignancy in HPV-negative neoplastic epithelial cells. HIV-mediated reductions in E-cadherin expression have been reported in both lung and gut epithelial cells (85, 86). HIV-induced EMT may be a common response shared by numerous HPV-negative neoplastic epithelial tissues in HIV-infected individuals.

The reduction in EMT and invasiveness of anal and cervical cells in response to inhibition of MAPK and TGF-β signaling, inhibition of vimentin expression, and restoration of E-cadherin suggest that there might be multiple approaches available to prevent HIV-associated acceleration of malignancy. Inhibition of vimentin by treatment with WFA or vimentin-specific siRNA initiated the normalization of neoplastic epithelium and promoted the conversion of various epithelial tumor cells to a MET phenotype (87–90). Restoration of E-cadherin expression in response to the COX-2 inhibitor, CXB, also resulted in a MET phenotype in head and neck squamous carcinoma cells (91). The induction of MET in primary tumors plays a critical role in blocking malignant cell migration and invasive activities.

**FIG 10:**
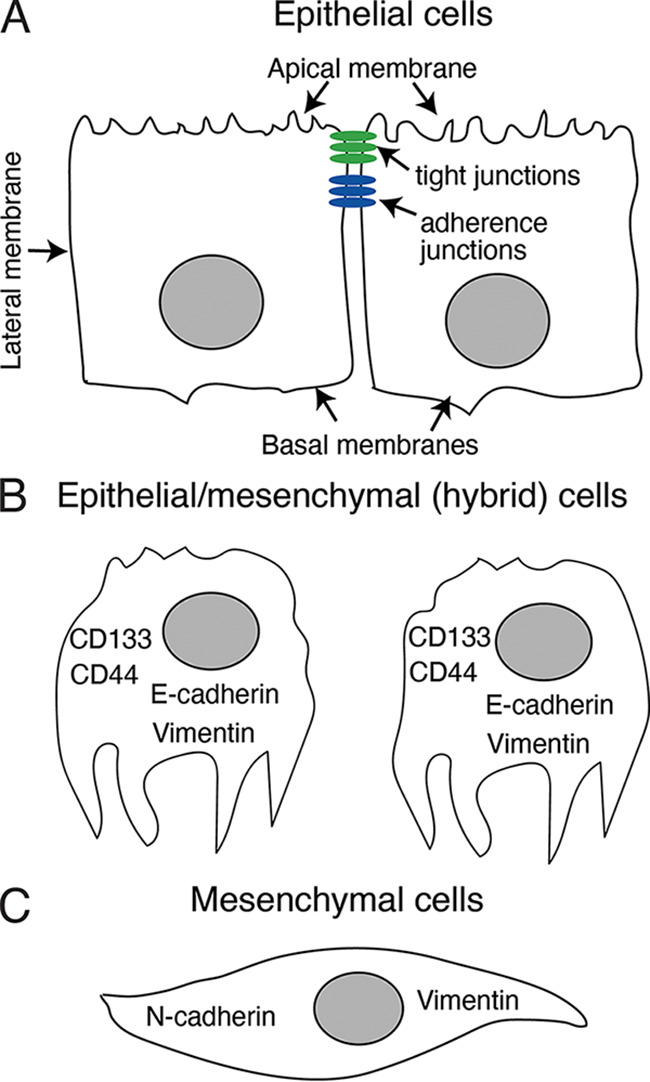
Model of HIV-induced EMT. (A) Genital and oropharyngeal mucosal epithelial cells have a polarized organization with well-developed tight and adherens junctions and distinct apical and basolateral membranes. (B) Interactions of HIV-1 cell-free virions and viral proteins gp120 and tat with epithelial cells result in activation of MAPK and TGF-β signaling. This ultimately leads to the induction of the EMT phenotype in which epithelial cells lose tight and adherens junctions, apicobasal polarity, and cobblestone-like morphology, and coexpress E-cadherin and vimentin. Cells displaying this hybrid phenotype also express stem cell markers and become CSCs with the potential to migrate through basement membranes and into the peripheral circulation, thereby generating invasive cancer and metastatic disease. (C) Further progression of EMT may lead to the loss of E-cadherin expression and a parallel upregulation of N-cadherin and vimentin expression as cells acquire a mesenchymal spindle-like morphology. Terminal mesenchymal cells are highly motile and invasive. Thus, this transition facilitates the spread of malignant cells within the tissues and organs. Collectively, our results support this model of HIV-associated acceleration of neoplastic processes in both HPV-infected and -uninfected premalignant/malignant mucosal epithelial cells.

In summary, we have shown that HIV-1 gp120 and tat proteins induce EMT in HPV-16 immortalized anal, cervical, and oral epithelial cells, leading to an increase in their invasive activity (Fig. 10). Moreover, HIV-1 proteins also increase the invasiveness of HPV-negative neoplastic oral cells. HIV-1 gp120- and tat-mediated induction of EMT features cells that coexpress both epithelial and mesenchymal markers. These cells can also express stem cell markers that may play a critical role in the formation of CSCs that contribute to metastases formed by neoplastic cells. Inhibition of TGF-β and MAPK signaling, together with the restoration of E-cadherin and suppression of vimentin expression blocked HIV-mediated increases in the invasive activity of neoplastic epithelial cells. Taken together, these results suggest several approaches that may be considered that will promote new strategies to block the progression of HIV-1-associated malignancies of both genital and oropharyngeal mucosal epithelial cells.

## MATERIALS AND METHODS

### Ethics statement.

This study was conducted according to the principles set forth by the Declaration of Helsinki and was approved by the Committee on Human Research of the University of California–San Francisco (IRB approval numbers 10–03277 and 19–27275).

### Viruses, viral proteins, and cells.

Laboratory-adapted dual-tropic (X4-R5) HIV-1_SF33_ and primary isolates R5-tropic HIV-1_SF170_ and X4-tropic HIV-1_92UG029_ were grown in peripheral blood mononuclear cells isolated from heparinized blood of HIV-1 negative healthy volunteers using a Ficoll-Paque Plus density gradient (Sigma). Cells were activated for 3 days with 2.5 μg/mL phytohemagglutinin (Sigma) and 1 μg/mL interleukin-2 (BD Biosciences). X4-tropic HIV-1_NL4-3_ and R5-tropic HIV-1_81A_ viruses were propagated in 293T cells. All viral stocks were purified using the Amicon Ultra-15 ultracentrifugation filtration system (Millipore). Viral stocks were titered based on their p24 concentrations with an HIV-1 p24 ELISA (PerkinElmer) according to the manufacturer’s instructions.

Recombinant HIV-1_Bal_ tat and its inactive form were purchased from ImmunoDX, LLC (Woburn, MA, USA). Inactive tat was created through alanine-substitution of both its basic arginine-rich domain (amino acids 49 to 57) as well as the integrin-binding RGD motif found at the C terminus (41–43). Purified recombinant gp120 proteins from HIV-1_BAL_, HIV-1_IIIB_, HIV-1_CN-54_, and HIV-1_96ZM651_ strains were provided by the U.S. National Institutes of Health (NIH) AIDS Reagent Program. HIV-1 gp120 was inactivated by incubation at 85°C for 30 min (92–94). All HIV proteins were stored at −80°C in the dark before use.

HPV-16-infected cervical CaSki and SiHa cells, oral SCC-47, and HPV-18-infected HeLa cell lines were obtained from American Type Culture Collection (ATCC, Manassas, VA, USA). HPV-negative cervical C-33 A, oral HSC-3, and pharyngeal Detroit-562 cancer cells were also obtained from ATCC. AKC-2 anal epithelial cells were established by transfecting HPV-negative primary anal keratinocytes with the pEF3-99 plasmid that contains the full-length 8kB HPV-16 genome with a single disruption of L1 ORF (32). HPV-16-immortalized oral HOK-16 epithelial cells were provided by Dr. No-Hee Park (University of California-Los Angeles).

### Treatment of epithelial cells with cell-free HIV virions and HIV proteins gp120 and tat and evaluation of EMT by H&E staining.

Cells plated at 3 × 10^4^/mL were treated with active tat, active gp120, inactive mutant tat, or heat-inactivated gp120, each at 10 ng/mL for 5 to 7 days as described in our previous publications (39, 40, 95). Cells were exposed to cell-free HIV-1 virions (10 ng/mL of p24) (10, 96, 97). HIV-1 virions were inactivated by exposure to UV irradiation at 100 mJ/cm^2^ (95). The culture medium was changed daily and fresh virus or proteins were added. One set of cells was treated with the MAPK inhibitor U0126 (10 μM; Sigma) or with the TGF-β1 inhibitor SB431542 (10 μM; Tocris Bioscience). Cells were also treated with Celecoxib (1 μM; Sigma) or WFA (0.1 μM; Selleckchem.com). WFA is a bioactive steroidal lactone isolated from *Withania somnifera*. A CellTiter-Glo luminescent cell viability assay (Promega) was performed to confirm the absence of cytotoxicity in response to the virus, tat, gp120, U0126, SB431542, CXB, or WFA. The assay was performed as described in the manufacturer’s instructions. Data were presented as relative luminescence units (RLU).

To counteract gp120-induced EMT and invasion by epithelial cells, gp120 from HIV-1_BAL_ was incubated (1 h at 37°C) with a pool of five anti-gp120 neutralizing antibodies, including 1 μg/mL each of b12, 2G12, F105, 39F, and ID6 (NIH AIDS Reagent Program) or with a pool of their isotype controls at the same concentrations. The gp120 protein together with neutralizing antibodies or isotype controls were added to cultures of tonsillar epithelial cells.

The cell culture medium was changed each day and included the addition of fresh proteins and antibodies. After 5 days when cell confluence reached 98% to 100%, cells were evaluated quantitatively for EMT with H&E staining. Cells that displayed a typical cobblestone shape were counted as epithelial cells, while cells with elongated spindle-like morphology and protrusions were counted as mesenchymal cells (i.e., EMT cells). Live EMT cells were also examined by phase-contrast inverted digital microscopy (Leica).

### Transfection of AKC-2 cells with siRNAs.

The siRNAs targeting HPV-16 E6 (sc-156008) and HPV-16 E7 (sc-270423) were purchased from Santa Cruz Biotechnology; nonspecific siRNA-A (sc-37007) was used as a negative control. Cells were transfected with E6+E7 siRNAs using lipid-based transfection reagents as previously described (32). HPV16 E7 silencing by siRNAs was confirmed at 72 h after transfection by a Western blot assay probed with antibodies E7-ED17 (Santa Cruz Biotechnologies) and E7-8C9 (Invitrogen) also as previously described (32).

### Collection of detached cells.

Cells were treated with active or inactive tat or gp120 (each at 10 ng/mL) for 5 days. Tissue culture medium with fresh proteins was changed every day. On day 5, the flasks containing these cells were then subjected to 5 min of gentle shaking on a rotary shaker (50 rpm). Floating (detached) cells were collected in 15-mL tubes and collected by centrifugation at 1,200 rpm for 10 min. Detached cells were then resuspended in complete medium and evaluated by counting on a hemocytometer. Unstained (live) cells were identified by trypan blue staining; only unstained cells were counted. Cells were then cultured in chamber slides or new plates/flasks for reattachment. The degree of reattachment was examined by phase-contrast inverted light microscopy starting on day 3 postseeding (Leica).

### Immunofluorescence assay.

For immunofluorescence assays, cells were fixed with 4% paraformaldehyde and 2% sucrose in phosphate-buffered saline (PBS) for 5 min, and then permeabilized with 0.01% Triton X-100 in 4% paraformaldehyde for an additional 5 min. Fixed cells were incubated with normal donkey serum (5%) in PBS to prevent nonspecific binding. Primary antibodies used in these studies included rabbit (Vector Laboratories) or goat anti-E-cadherin antibodies (R&D Systems), goat anti-vimentin (Millipore), and rabbit-anti-pan keratin (Life Technologies). CD133 and CD44 were detected with antigen-specific rabbit polyclonal antibodies (Abcam). Isotype-matched antibodies were used as negative controls to confirm the specificity of each primary antibody. Cells were incubated with primary antibodies for 1.5 h. Secondary antibodies used in this assay include Dylight 488, Dylight 594 (Vector Laboratories), and Alexa Fluor 594 (Jackson ImmunoResearch Laboratories). Cell nuclei were counterstained with DAPI (Molecular Probes). Images were captured with a Nikon Eclipse E400 fluorescence microscope (Nikon) or Leica SP5 confocal microscopy (Leica Microsystems). Quantitative analysis of EMT was undertaken by enumeration of the E-cadherin- and vimentin-expressing cells relative to the total number of cells per field in 10 individual and randomly chosen fields on each slide. Cell counting was performed independently by three investigators (K.L., W.M., R.P.) using NIH ImageJ software.

### Western blot assay.

Cells were extracted with 1.0% Triton X-100 buffer (150 mM NaCl and 10 mM Tris/HCl, pH 8.0), with protease inhibitors (Sigma). Proteins were separated on a 4%- to 20%-gradient sodium dodecyl sulfate-polyacrylamide gel and transferred to a membrane (GE Healthcare). The blots were probed with the following primary antibodies: rabbit anti-E-cadherin (R&D Systems); rabbit anti-N-cadherin, rabbit anti-vimentin, and rabbit anti-Snail (Cell Signaling); monoclonal mouse anti-TGF-β1 (Thermo Fisher Scientific and Abcam); rabbit anti-total ERK1/2 and rabbit anti-phosphorylated ERK1/2. HPV-16 E7 was detected by using antibodies E7-ED17 (Santa Cruz Biotechnologies) and E7-8C9 (Invitrogen) as previously described (32). Equivalent protein loading was confirmed by probing with anti-beta-actin (Ambio).

### Invasion assay.

*In vitro* invasion assays were performed using the Collagen Cell Invasion Assay-Colorimetric (8 μM; EMD Millipore) according to the manufacturer’s protocol and as previously described in our laboratory (10, 32). Cells were cultured with cell-free HIV-1 virions, gp120, and/or tat proteins or their inactive controls for 5 to 7 days. Cells (5 × 10^4^ in Dulbecco’s modified Eagle medium) were then used to seed each of the collagen-coated inserts. Fetal bovine serum (10%) was added to the lower chamber as a chemoattractant. Cell migration and invasion of the collagen inserts were evaluated 24 h later by crystal violet staining followed by microscopy. Cell migration/invasion under various experimental conditions was examined quantitatively by counting the number of crystal-violet-stained cells in 10 randomly selected fields. The data are presented as the average number of cells per field. Data are shown as means ± SD from at least 10 independent microscopic fields.

### Statistical analysis.

Statistical comparisons were performed using two-tailed Student’s t-tests. A *P* value of <0.05 was considered to be statistically significant. Results are expressed as mean ± SD unless otherwise indicated.
