# Supplementary material for: HIV-1 Proteins gp120 and Tat Promote Epithelial-Mesenchymal Transition and Invasiveness of HPV-Positive and HPV-Negative Neoplastic Genital and Oral Epithelial Cells
Source: Microbiol Spectr. 2022 Oct 31;10(6):e03622-22. doi: 10.1128/spectrum.03622-22 (PMC9770004; doi:10.1128/spectrum.03622-22)
Supplement: Supplemental file 1 — Fig. S1 to S5. Download spectrum.03622-22-s0001.pdf, PDF file, 2.6 MB [file spectrum.03622-22-s0001.pdf]

Supporting Information

Supplementary figures

S1 Fig. Quantitative analysis of E-cadherin and vimentin expression and EMT in AKC-2 and CaSki cells. (A) Cells were grown under similar conditions for two months. Every two weeks, cells were sampled and used to seed chamber slides for quantitative analysis of E-cadherin and vimentin expression. (B) Percentage of E-cadherin and vimentin co-expressing cells identified by immunofluorescence staining. (C) The percentage of EMT cells (cells presenting with a spindle shape) was determined quantitatively from H&E-stained preparations. Data are shown as means ± SD from at least 10 independent microscopic fields; *$p<0.05$, **$p<0.01$, ***$p<0.001$ compared to the values shown at the following time point.

S2 Fig. HIV proteins gp120 and tat induce an EMT in AKC-2 and CaSki cells. (A) AKC-2 and (B) CaSki cells are left untreated (control) or treated with inactive and active forms of HIV proteins tat and gp120 (each at 10 ng/ml). After five days, live cells were examined by phase-contrast microscopy. Representative phase-contrast images are shown. Magnification: x100.

S3 Fig. Quantitative analysis of expression of EMT markers in AKC-2 and CaSki cells by Western blot. To quantify protein expression in AKC-2 and CaSki cells in response to HIV-gp120 or tat by Western blot (shown in Fig. 2D), the integrated

densities of pixels in each of the immunoreactive protein bands (E-cadherin, vimentin, Snail, and N-cadherin) were determined by ImageJ software. These findings are presented as a bar graph.

S4 Fig. Inactivation of HIV-1 gp120 by neutralizing antibodies. HIV-1 gp120 preincubated with a pool of neutralizing antibodies (b12, 2G12, F105, 39F, and ID6) (+ab) or isotype controls (+isotype) for 30 min at 37˚C was added to AKC-2 cells in culture. After five days, the cells were fixed and hematoxylin and eosin-stained for quantitative evaluation of mesenchymal cells. Data shown are the means ± SD for ten independent fields; ****p*<0.001 for antigen-specific *vs* isotype control antibodies. Data are representative of three independent experiments.

S5 Fig. Evaluation of AKC-2 and CaSki cell viability in response to various inhibitors. AKC-2 and CaSki cells were untreated (control) or treated for five days with MAPK (UO126), TGF-β1 (SB431542), vimentin (WFA), or cyclooxygenase-2 (celecoxib) inhibitors. Viability was assessed using a CellTiter-Glo® luminescent cell viability assay. Data are representative of two independent experiments and are shown as means ± SD (n=10). RLU, relative luminescence units.

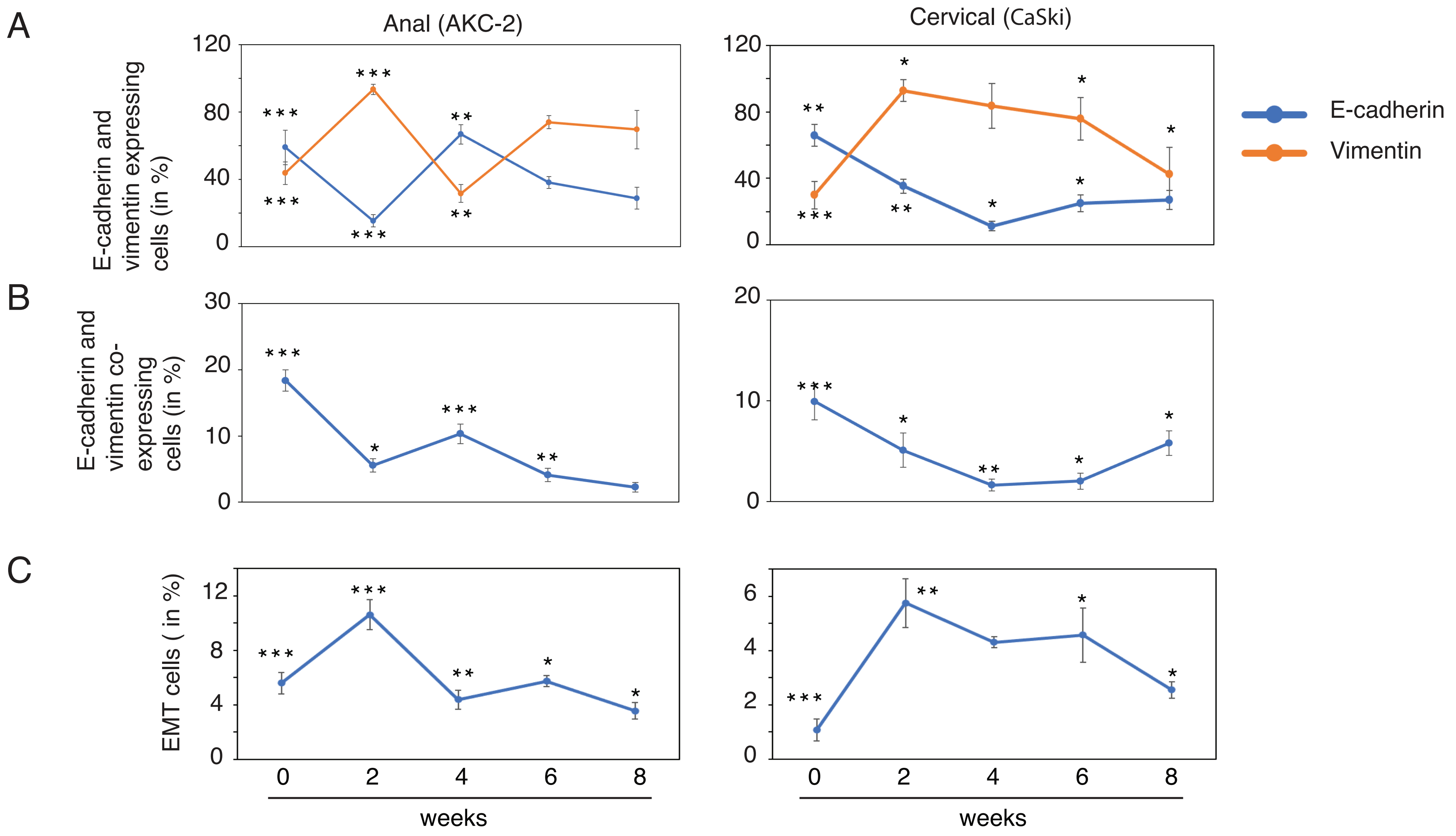
A
B
C
Anal (AKC-2)
Cervical (CaSki)
E-cadherin and vimentin expressing cells (in %)
E-cadherin and vimentin co-expressing cells (in %)
EMT cells (in %)
E-cadherin
Vimentin
weeks
weeks

A

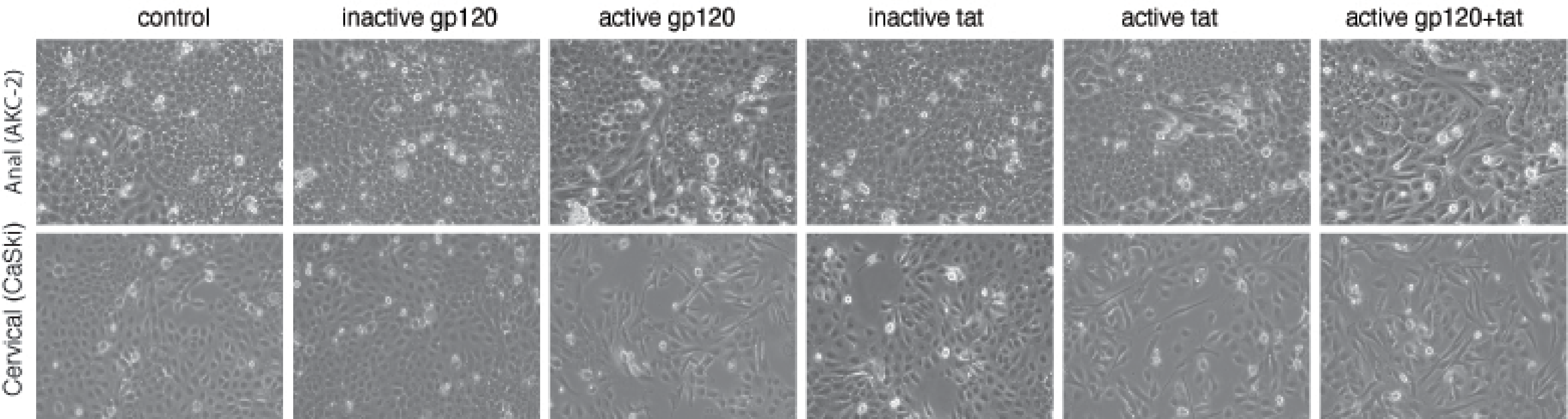
control
inactive gp120
active gp120
inactive tat
active tat
active gp120+tat
Anal (AKC-2)
Cervical (CaSki)

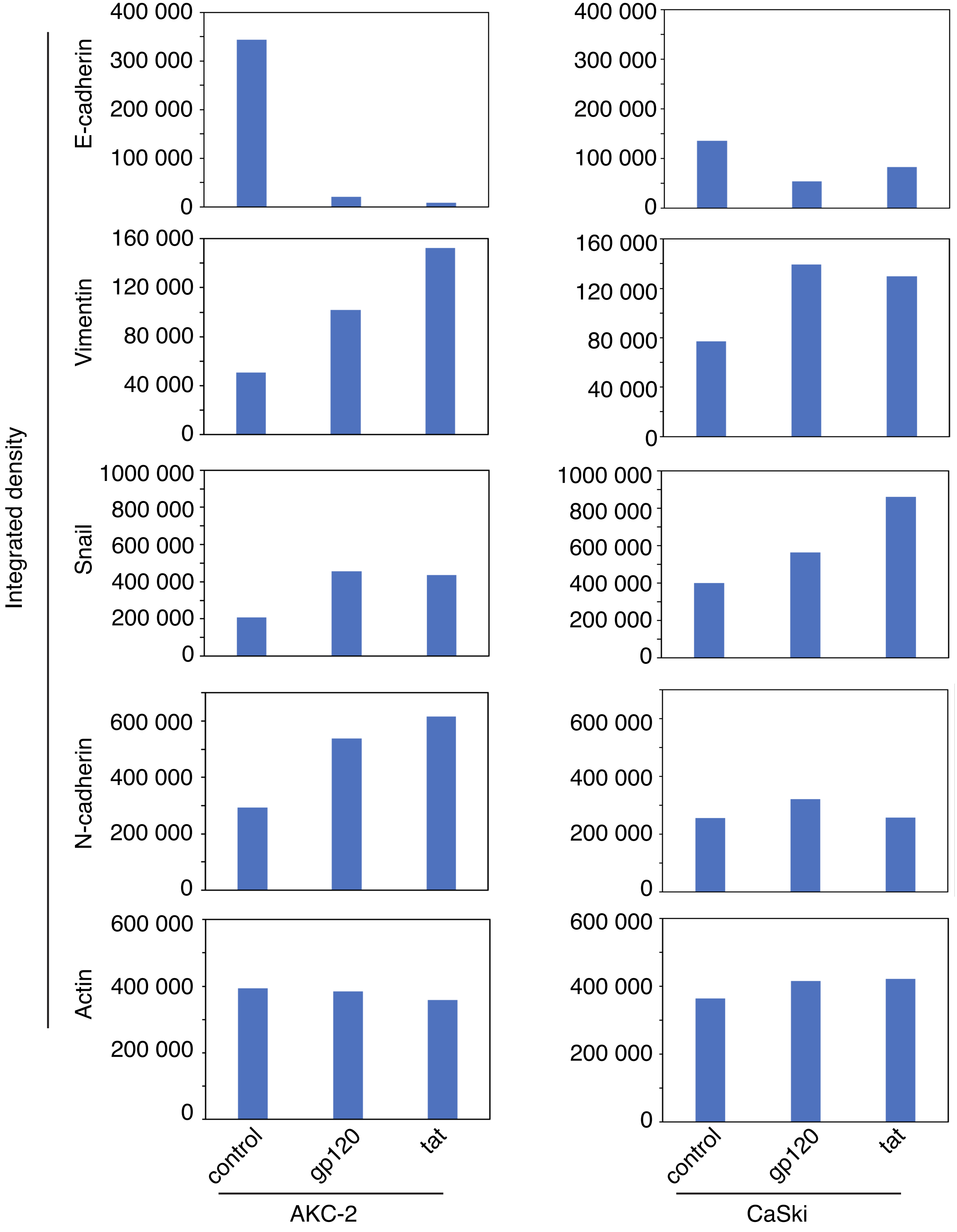
Integrated density
E-cadherin
Vimentin
Snail
N-cadherin
Actin
400 000
300 000
200 000
100 000
0
160 000
120 000
80 000
40 000
0
1000 000
800 000
600 000
400 000
200 000
0
600 000
400 000
200 000
0
control
gp120
tat
AKC-2
CaSki

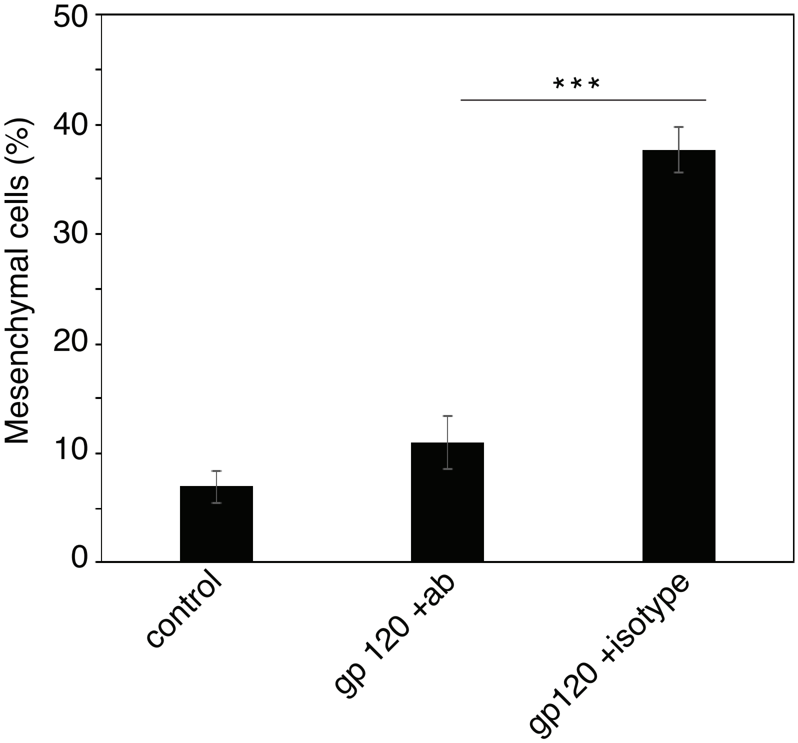

50
40
30
20
10
0
Mesenchymal cells (%)
***
control
gp 120 +ab
gp120 +isotype

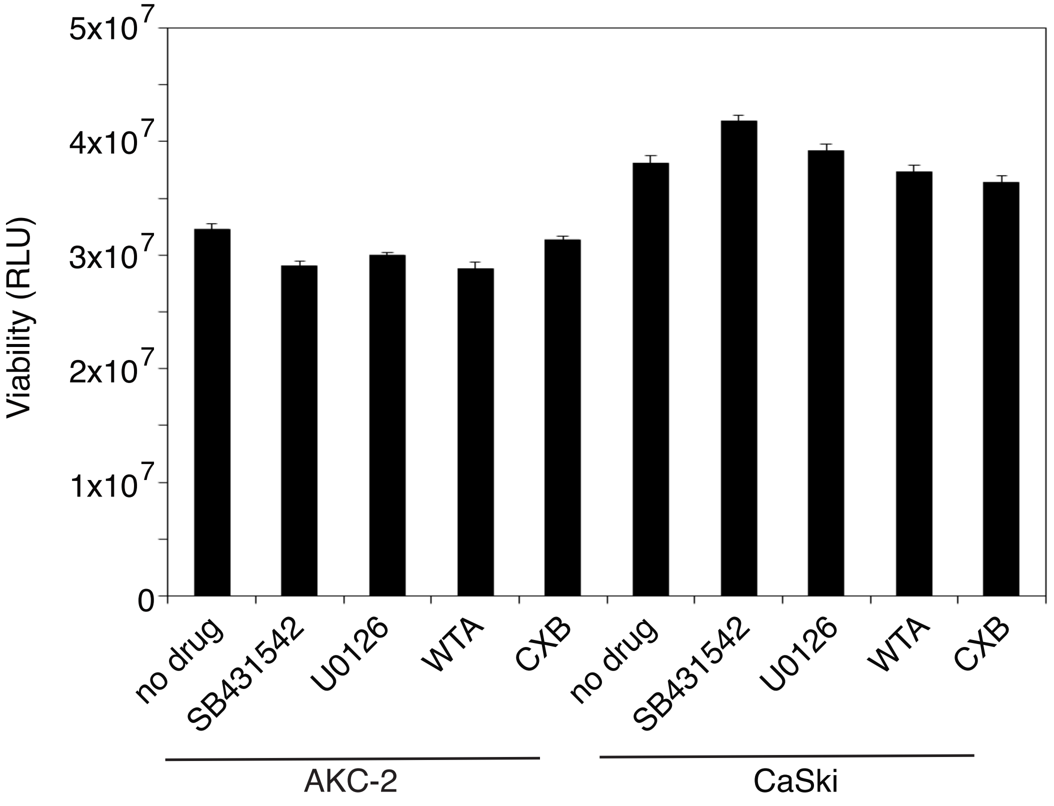
Viability (RLU)
5x10^7
4x10^7
3x10^7
2x10^7
1x10^7
0
no drug
SB431542
U0126
WTA
CXB
no drug
SB431542
U0126
WTA
CXB
AKC-2
CaSki
